# Supplementary material for: Protocol for a feasibility randomised controlled trial of a musculoskeletal exercise intervention versus usual care for children with haemophilia
Source: BMJ Open. 2019 Aug 1;9(8):e029474. doi: 10.1136/bmjopen-2019-029474 (PMC6688743; doi:10.1136/bmjopen-2019-029474)
Supplement: Author's manuscript [file bmjopen-2019-029474.draft_revisions.pdf]

BMJ Open is committed to open peer review. As part of this commitment we make the peer review history of every article we publish publicly available.

When an article is published we post the peer reviewers' comments and the authors' responses online. We also post the versions of the paper that were used during peer review. These are the versions that the peer review comments apply to.

The versions of the paper that follow are the versions that were submitted during the peer review process. They are not the versions of record or the final published versions. They should not be cited or distributed as the published version of this manuscript.

BMJ Open is an open access journal and the full, final, typeset and author-corrected version of record of the manuscript is available on our site with no access controls, subscription charges or pay-per-view fees (http://bmjopen.bmj.com).

If you have any questions on BMJ Open's open peer review process please email info.bmjopen@bmj.com

BMJ Open

Protocol for a feasibility randomised controlled trial of a musculoskeletal exercise intervention versus usual care for children with haemophilia

Journal:	*BMJ Open*
Manuscript ID	bmjopen-2019-029474
Article Type:	Protocol
Date Submitted by the Author:	29-Jan-2019

Complete List of Authors:	Hashem, Ferhana; University of Kent, Centre for Health Service Studies<br>Bladen, Melanie; Great Ormond Street Hospital For Children NHS Foundation Trust, Haemophilia Centre<br>Carroll, Liz; Haemophilia Society<br>Dodd, Charlene; East Kent Hospitals University NHS Foundation Trust, Haemophilia Centre<br>Drechsler, Wendy; King's College London, School of Population Health & Environmental Sciences<br>Lowery, David; University of Kent, Centre for Health Service Studies<br>Patel, Vishal; Barts Health NHS Trust, Haemophilia Centre<br>Pellatt-Higgins, T; University of Kent, Centre for Health Services studies<br>Saloniki, Eirini; University of Kent, Centre for Health Service Studies<br>Stephensen, David; East Kent Hospitals University NHS Foundation Trust, Haemophilia Centre; Barts Health NHS Trust, Haemophilia Centre

Keywords:	haemophilia, physiotherapy, exercise, muscle strength, physical function

Note: The following files were submitted by the author for peer review, but cannot be converted to PDF. You must view these files (e.g. movies) online.
DOLPHIN Feasibility RCT - SPIRIT_checklist.docx

SCHOLARONE™
Manuscripts

Protocol for a feasibility randomised controlled trial of a musculoskeletal exercise intervention versus usual care for children with haemophilia

Hashem F[1],Bladen M[2], Carroll L[3], Dodd C[5], Drechsler WI[4], Lowery D[1], Patel V[6], Pellatt-Higgins T[1], Saloniki E-C[1,7], Stephensen D[5,6]

[1]University of Kent, Centre for Health Service Studies, Canterbury, United Kingdom

[2]Great Ormond Street Hospital For Children NHS Foundation Trust, Haemophilia Centre, London, United Kingdom

Institute of Child Health, University College London, London, United Kingdom

[3]Haemophilia Society UK, London, United Kingdom

[4]Kings College London, School of Population Health & Environmental Sciences, London, United Kingdom

[5]East Kent Hospitals University NHS Trust, Haemophilia and Thrombosis Centre, Canterbury, United Kingdom

[6]Royal London Hospital, Haemophilia Centre, London, United Kingdom

[7]University of Kent, Personal Social Services Research Unit, Canterbury, United Kingdom

Corresponding Author: Dr David Stephensen, East Kent Hospitals University NHS Trust, Haemophilia and Thrombosis Centre, Canterbury, United Kingdom. david.stephensen@nhs.net

Key Words: Haemophilia, Physiotherapy, Exercise, Muscle strength, Physical function

Abstract word count: 277 words

Manuscript word count: 3283 words (excluding Figure and References)

For peer review only - http://bmjopen.bmj.com/site/about/guidelines.xhtml

ABSTRACT

Introduction: Haemophilia is a rare, inherited disorder in which blood does not clot normally, resulting in bleeding into joints and muscles. Long-term consequence is disabling joint pain, stiffness, muscle weakness, atrophy and reduced mobility. The purpose of this proposed feasibility of a randomised controlled trial (RCT) is to test the feasibility of an age-appropriate physiotherapy intervention designed to improve muscle strength, posture and the way boys use their joints during walking and everyday activities.

Methods and analysis: A small-scale two-centre RCT of a 12-week muscle strengthening exercise intervention versus usual care for young children with haemophilia will be conducted. Primary outcomes will be safety and adherence to the exercise intervention. Secondary outcomes will include: recruitment, retention and adverse event rates, clinical data, muscle strength, joint biomechanics and foot loading patterns during walking, six-minute timed walk, timed-up-and-down-stairs, EQ-5D-Y, participants' perceptions of the study, training requirements and relevant costs. Recruitment, follow-up, safety and adherence rates will be described as percentages. Participant diary and interview data will be analysed using a framework analysis. Demographic and disease variable distributions will be analysed for descriptive purposes and co-variant analysis. Estimates of differences between treatment arms (adjusted for baseline), and 75% and 95% confidence intervals will be calculated.

Ethics and Dissemination: The study has ethical approval from the London - Fulham Research Ethics Committee (17/LO/2043) as well as Health Research Authority approval. As well as informing the design of the definitive trial, results of this study will be presented at local, national and international physiotherapy and haemophilia meetings as well as manuscripts submitted to peer-reviewed journals. We will also share the main findings of the study to all participants and the Haemophilia Society.

For peer review only

ARTICLE SUMMARY

Strengths and limitations of this study

At the end of this study we will not be able to answer the question "Does exercises help improve the long term health of children with haemophilia?" However, we will know whether such a study could be undertaken and the best way to deliver the intervention. The information learned will be used to determine if the exercises are safe and can be completed by children with haemophilia. It will also inform estimation of the sample size for a definitive trial and what costs might be incurred by doing the exercises.

- This study focuses on the feasibility of a planned multi-centre randomised controlled trial of a muscle strengthening exercise intervention versus usual care in children with haemophilia
- It is impossible to blind those in receipt and those delivering the intervention as to the group allocation, but we will blind the assessor as to group allocation
- The study will determine whether recruitment and adherence strategies are appropriate.

For peer review only

INTRODUCTION

Haemophilia is a rare, inherited disorder in which blood does not clot normally. Repeated spontaneous and traumatic bleeding into joints and muscles is the result; the ankle joint being the most common.[1] Long-term consequence is chronic arthropathy, a pattern of disabling joint pain, stiffness, muscle weakness, atrophy and reduced mobility.[2-4] The introduction of prophylaxis with clotting factor concentrates has reduced the number of bleeding episodes experienced by children, changing their lifestyle and long term outlook.[5-6] However, evidence indicates that despite prophylaxis, boys still bleed 1-2 times per year, with relatively few bleeding episodes being required to initiate bone destruction, and in some cases, articular erosion is evident with no history of joint bleeding.[1,7-8] Furthermore, children are more susceptible to blood induced damage than adults and, once established is progressive and irreversible.[9]

It is well established that muscle weakness is associated with haemophilic arthropathy in adults[4] and it is now becoming apparent that muscle strength is reduced in children, prior to the onset of clinical arthropathy.[10] In a study of 10 adolescent and adult haemophiliacs with a history of unilateral knee bleeding, Falk *et al.* (2005) found that strength of the knee extensors and flexors in haemophilic children were significantly weaker than those of the age-matched controls.[11-12] More recently, Stephensen *et al.* (2012) observed that despite normal clinical examinations and comparable levels of physical activity, young haemophilic boys with a history of ankle joint haemorrhage had significantly reduced dynamic muscle strength together with atrophy of the knee extensors, ankle dorsi- and plantarflexors when compared to age and size-matched typically developing peers.[10] Furthermore, muscle weakness of the leg is strongly correlated to muscle atrophy, reduced walking distances, slower ascent and descent of stairs, as well as reduced ankle joint motion, greater knee flexion motion and forces at the knee and ankle joint during walking.[13-14]

Therapeutic exercise is an important component of the management of other forms of arthropathy (e.g. osteoarthritis, rheumatoid arthritis) [15] and it would appear logical that exercise would be effective for people with haemophilia. There is growing evidence of the benefits of incorporating muscle strengthening exercise in their management of arthropathy. The rationale for this is that increases in muscular strength and postural control might improve motor performance and cardiovascular fitness, limit exaggerated end-range joint movement, and

For peer review only - http://bmjopen.bmj.com/site/about/guidelines.xhtml

promote optimal transfer of weight-bearing forces through joints thereby minimising muscle imbalance, synovial impingement and associated haemarthroses or synovitis.[16] A recent Cochrane Review evaluating the safety and effectiveness of exercise for people with haemophilia reported four randomly controlled studies of an exercise intervention in children with the condition and concluded the studies were of low or very low quality, due to small sample sizes and potential bias.[17] Furthermore, no paediatric study compared a muscle strengthening intervention to a control group or intervention without muscle strengthening exercises. Several studies compared two multi-component exercise interventions that included broad muscle strengthening as well as treadmill walking and cycle ergometry and it is not possible to determine the effect of muscle strengthening on muscle strength or its effect on physical function.[18-19] Furthermore, pre-adolescent and adolescent boys aged between 10 and 14 were included in these studies, and it is not known if the groups were matched for pubertal status.

Despite the apparent benefit, there is a lack of robust evidence to determine whether muscle strengthening exercise can improve or negatively affect outcomes for young children with haemophilia. The purpose of this proposed feasibility RCT is to test the feasibility of an age-appropriate physiotherapy intervention designed to improve muscle strength, posture and the way boys use their joints during walking and everyday activities.

METHODS AND ANALYSIS

The protocol version for this feasibility study is Version 2 – 19/12/2017. The Standard Protocol Items: Recommendations for Interventional Trials (SPIRIT) guidelines[20] have been used to report the study protocol.

Participants

Participants will be invited from two haemophilia centres. Twenty boys with severe or moderate haemophilia with or without inhibitors, aged 6-11 years will be recruited. Exclusion criteria are: past history of fracture or trauma to the lower limb; orthopaedic surgery; acquired brain injury or any other disturbance of the central nervous system; joint or muscle bleed in the lower limb in the past 6 weeks; presence of lower limb pain or unable to fully comply with verbal instructions.

For peer review only - http://bmjopen.bmj.com/site/about/guidelines.xhtml

Participants will be identified from each Haemophilia Centre's database of registered patients. The parents or guardians of eligible participants will be approached initially by the principle investigator at each site via telephone to provide a brief verbal explanation of the research study and ask if they would be happy to receive a patient information sheet. Should they be interested in participating in the study they will receive, by post, the patient information sheet (one for parents and a separate age appropriate version for children) at least one week prior to their next clinic appointment. This will allow for a 'cooling-off' period of a minimum of one week for the potential participants. The researcher will then be available for questions by telephone or at the participant's next clinic appointment.

At their clinic appointment, the investigator will meet with the participant and parent/ legal guardian to complete the consent form as appropriate. The investigator will further explain the study procedures, why they are being asked to consider including their child in this study and the associated risks and benefits of being included. They will then be given an opportunity to ask any further questions. The child/ parent/ legal guardian will then be asked to confirm if the potential participant agrees to take part in the study. If consent is agreed a parental consent form will be completed by the parent/ guardian and a copy will be provided. If at any point during the study process the parent/guardian does not consent to inclusion in the study then any data collected will be destroyed. Any decision to refuse or withdraw from the study will in no way influence the services that they receive. Once the child's parent/guardian has given consent, the child must also indicate that they do not object to participating in the research activity. This participant 'assent' will be sought.

Study design

Following written informed consent and screening of eligibility criteria, randomisation of eligible participants for the study will be performed with the online randomisation service http://sealedenvelope.com/. Utilising the secure, centralised and independent service, participants will be randomly allocated into one of two groups (Treatment Group 1 will receive a 12-week muscle strengthening exercise intervention; Treatment Group 2 will receive usual physiotherapy care for 12 weeks) on a ratio of 1:1. Allocation will be stratified by centre to ensure the same numbers of participants at each site are allocated to the two groups. The un-blinded principal investigator at each site will be issued with a password to access the service to create a new randomisation for the study participants. Those allocated to Treatment Group 2

For peer review only - http://bmjopen.bmj.com/site/about/guidelines.xhtml

will be offered the intervention after their end of study follow-up assessment. Data will be collected prior to and immediately post intervention (Figure 1).

It is impossible to blind those in receipt and those delivering the intervention as to the group allocation. However, it will be possible – although perhaps challenging - to blind the assessor as to group allocation. Consequently, we will attempt a single blind approach in which the participant and physiotherapists delivering the intervention will be encouraged to withhold their group allocation from the assessors collecting data. We will use a range of strategies, including: instruction in the participant literature; intervention protocol and with frequent verbal prompts. In order to assess the efficacy of blinding, we will ask assessors to record which group they think participants have been allocated to at the end of each assessment. This will be presented as a binary forced choice. There are no circumstances under which it would be necessary to un-blind assessors.

<u>Intervention</u>

The intervention, developed by expert clinicians and patients utilising a modified nominal group technique is a 24-session, 12-week programme designed as 2-week progressive levels (intensity and or load) with no more than ten exercises in each session, taking 25-30 minutes to complete. The intervention aims to master movement control and emphasizes body-weight strength development. Pictures of the exercises and instructions for each phase will be provided in 2-week exercise diaries. Participants will be asked to complete the exercises twice per week; once with the physiotherapist who will visit the participant at home, and once supervised by their parents/ guardian. Completion of exercises, along with treatment regime, any adverse events and comments in relation to the exercise programme will be recorded in the exercise diary. They will also record participation in physical activities, including type, time and intensity. According to predetermined criteria (safe and competent), the physiotherapist will determine if it is appropriate for the participant to progress to the next exercise level. On return of the exercise diary every 2 weeks, participants will be provided with a gift voucher.

<u>Usual care</u>

Participants will continue to receive their usual care as recommended and prescribed by the haematologist, physiotherapist and other members of the healthcare team. This will include factor concentrate treatment, any physiotherapy or surgical intervention or advice that is recommended by the clinical care team. The researchers will not make or initiate any changes

For peer review only - http://bmjopen.bmj.com/site/about/guidelines.xhtml

to routine treatment. Any change to or initiation of new treatment or intervention will be recorded and the treating clinician will determine whether it is appropriate for the participant to continue in the study. Participants will be provided with a diary to record participation in physical activities, including type, time and intensity. Participants will be telephoned weekly to encourage dairy completion. On return of the diary every 2 weeks, participants will be provided with a gift voucher.

<u>Outcome measures</u>

The primary outcome measures are:

- Safety will be evaluated using annualised bleeding rates (ABR), coagulation factor usage and reported adverse events and reactions associated with the intervention
- Adherence to the exercise intervention will be evaluated by the number of exercise session completed as a percentage of the prescribed exercise sessions. Data will be extracted from participant diaries

Secondary outcome measures are:

- Recruitment rate determined as the participants who volunteer to participate in the study as a percentage of those that are invited/ eligible
- Willingness to be randomised; evaluated at the end of study through semi-structured interviews that will be audio-recorded and be transcribed by a member of the research team
- Participant's acceptability of the trial and intervention: using semi-structured interviews, conducted at the completion of the study, the participants will be asked their views on acceptability of the trial and intervention, recruitment and exercise adherence. The interview will be audio-recorded and be transcribed by a member of the research team
- Training required to deliver the intervention and collect outcome data at study visits: using semi-structured interviews, conducted at the completion of the study, the physiotherapists will be asked their views on acceptability of the trial and intervention, recruitment and exercise adherence. They will also be asked about the training requirements for delivering the intervention as well as the appropriateness of the outcomes piloted in terms of training, length of time to complete and clinical value of the data. The interview will be audio-recorded and be transcribed by a member of the research team
- Haemophilia Joint Health Score (HJHS) – a clinical examination of swelling, muscle atrophy, strength, joint crepitus, range of motion and pain. The HJHS is a standardised

For peer review only - http://bmjopen.bmj.com/site/about/guidelines.xhtml

multi-item assessment, currently used in clinic as part of routine care to record musculoskeletal health[21]

- Haemophilic synovitis and arthropathy – The presence and severity of signs of haemophilic synovitis and arthropathy will be evaluated using the Haemophilia Early Arthropathy Detection with Ultrasound (HEAD-US) score. The HEAD-US protocol is a standardised scanning protocol, used to identify the presence and severity of synovitis, articular cartilage and chondral bone defects in the ankle, knee and elbow joint. The scanning procedure takes approximately 2-3 minutes per joint and we have recently shown good inter-rater repeatability of this protocol when performed by physiotherapists[22-23]
- Muscle strength of the knee extensors and ankle dorsi- and plantarflexors – Maximum torque will be recorded with a hand held dynamometer (Manual Muscle Tester, Lafayette Instrument, USA) using our standardised protocol with excellent test-retest repeatability[14]
- Joint biomechanics and motion during walking – Temporal spatial and three-dimensional (3D) joint motion during gait will be recorded using an inertial magnetic measurement gait analysis system (MVN BIOMECH Awinda, Xsens Technologies, Netherlands). Sensor units will be attached to the feet, lower legs, thighs, and pelvis with elastic straps. Motion of the sensors will be used to calculate temporal spatial and 3D lower limb joint motion
- Static and dynamic foot loading patterns – will be evaluated with a plantar pressure platform (HR Mat, Tekscan, USA). Centre of pressure motion in respect of distance and direction travelled, together with contact time as a measure of foot symmetry of foot loading for various foot segments (heel, midfoot and forefoot) along with distribution of peak pressure and pressure-time integrals will be evaluated
- Six-minute timed walk (6MTW) – Distance walked in 6 minutes. Two marker cones will be placed 30 metres apart along a corridor and each subject will be asked to walk back and forth between the cones as fast as they can for 6 minutes.[14]
- Timed up and down stairs (TUDS) – Time taken to ascend and descend a flight of 12 steps. The children will stand 30-centremetres from the bottom step and asked to walk quickly but safely up the stairs, turn around on the top step and come all the way down until both feet land on the bottom step. They will be instructed to step on every step, use the hand rail if they want and not to run.[14]
- EQ-5D-Y – a five dimension (mobility, self-care, usual activities, pain/discomfort, anxiety/depression) questionnaire measuring health-related quality of life.[24]

For peer review only - http://bmjopen.bmj.com/site/about/guidelines.xhtml

- Costs relevant to the study – intervention costs, health and social care costs, out-of-pocket expenses and time off work costs.

All data collected related to the study will be confidential and anonymous. The participant will be allocated a randomised participant number code and all data collected will relate to this code. A record of which code belongs to which subject will be stored in a locked cabinet separate from the study data. Only the principle investigator (who will be a member of the participant's direct care team) will have access to this documentation. The direct care team and the principal investigator will have access to participant's personal and identifiable information during the study. Consent will be sought by the lead researcher for the research team to have access to this personal information.

Analysis methods

Recruitment, follow-up, safety and adherence rates will be described as percentages. Participant diary and interview data will be analysed to identify factors that influence these rates and acceptability of the trial using a framework analysis. Interview data from physiotherapists will be analysed using a framework analysis to determine training required to deliver the intervention and collect outcome data. Feasibility of collecting healthcare cost data will be explored along with the resource consequences. Costs will be analysed and reported using descriptive statistics. Demographic and disease variable distributions will be analysed for descriptive purposes and co-variant analysis. Group mean and standard deviation (median and inter-quartile range where appropriate) will be calculated and reported. Change from baseline data will be compared between groups at all study time points using analysis of covariance with baseline as a covariate and a factor for centre. Estimates of differences between treatment arms in the changes from baseline (adjusted for baseline), and 75% and 95% confidence intervals will be calculated. Group mean and standard deviation (median and inter-quartile range where appropriate) will be calculated pre and post intervention. These data will allow us to estimate the potential effect of the intervention and provide estimates of variability to inform the sample size required for a full RCT, as well as to refine the outcome measures by evaluating their responsiveness to changes in musculoskeletal health.

For peer review only - http://bmjopen.bmj.com/site/about/guidelines.xhtml

Patient and Public Involvement

Ten specialist haemophilia physiotherapists in the United Kingdom plus those experienced in sports injury were invited to take part in a Nominal Group Technique (NGT) consensus discussion to share their experience and expertise to contribute to the development of the exercise intervention. Patients' priorities, experience, and preferences informed the study design, recruitment as well as the design and delivery of the intervention. The exercise programme was demonstrated to five boys with haemophilia and their parents who were members of the Haemophilia Society, national UK patient organisation. The parents and children were asked about what they thought about the exercises and whether they could undertake them on a regular basis, where they thought the best place was for undertaking them, and how they would like to receive the information on the exercise programme. Parents and children were then asked questions about how they would feel about taking part in study testing the benefits of the exercises, issues around being allocated randomly into study groups, and then questions about what would encourage the children to continue on the exercise programme with discussions focusing upon types of rewards and incentivisation.

Management and oversight of the study

East Kent Hospital University Foundation Trust is the study sponsor. They have overall responsibility for governance of the study and have no direct role in study design, data collection, management, analysis, interpretation of data and reports and publications that arise from the study.

A Research Monitoring Committee (RMC) has been established and will meet six-monthly after the study has commenced. The RMC will be responsible for monitoring milestones and targets, data, safety, recruitment, reviewing and interpreting the results. The RMC will be chaired by an independent chair from outside the research team group. Additional meetings may be called to review any serious adverse events (SAE), adverse events (AE) and adverse reactions (AR). The Chief Investigator will: co-ordinate day to day running of the research; complete ethical approvals; communicate with recruitment sites; complete training of physiotherapists to deliver the intervention; undertake research initiation and monitoring site visits; co-ordinate data analysis and interpretation. The Chief Investigator will notify the RMC in the case of AEs and ARs. The Principal Investigator at each site will be the physiotherapist delivering the intervention. They will be responsible for recruitment and screening participants according to the inclusion and exclusion criteria, reporting AEs and ARs and local governance requirements.

For peer review only - http://bmjopen.bmj.com/site/about/guidelines.xhtml

ETHICS AND DISSEMINATION

Favourable ethical approval was obtained from the Health Research Authority and London - Fulham Research Ethics Committee and (17/LO/2043).

Study newsletters will be published by the RMC and available on the website of the Haemophilia Society. The results of this feasibility study will inform the design of a definitive RCT to test whether muscle strengthening can benefit musculoskeletal health in children with haemophilia, including whether such a study can be achieved and the best way of completing it. The results of the feasibility study will be presented at local, national and international physiotherapy and haemophilia meetings as well as manuscripts submitted to peer-reviewed journals. The main findings of the study will be shared to all participants and the Haemophilia Society.

AUTHORS CONTRIBUTION

DS, MB and WD contributed to the inception of the study. All co-authors contributed to the design of the protocol. DS, MB, WD, CD and VP contributed to the design of the intervention. FH and DS drafted the manuscript and all co-authors critically revised the manuscript.

ACKNOWLEDGEMENTS

The authors would like to acknowledge the contribution of the academic experts and specialist physiotherapists (Wendy Drechsler, Melanie Bladen, Danny Armitage, Trupti Bhandari, Lisa Geuran, Hannah Harbidge, Nicola Hubert, Ann McCarthy, Julie Sparrow, Linda Walsh), the children and parents of the Haemophilia Society for sharing their priorities, experience, and preferences that informed the study design, recruitment as well as the design and delivery of the intervention.

FUNDING:

We gratefully acknowledge the National Institute for Health Research – Research for Patient Benefit Programme for providing funds to support this project (grant number PB-PG-0215-36091).

CONFLICT OF INTEREST

The authors declare no conflicts of interest.

For peer review only - http://bmjopen.bmj.com/site/about/guidelines.xhtml

REFERENCES

1. Stephensen D, Tait R, Brodie N et al. Changing patterns of bleeding in patients with severe haemophilia A. Haemophilia 2009; 15: 1210–4.
2. Roosendaal G, van Rinsum AC, Vianen ME, et al. Haemophilic arthropathy resembles degenerative rather than inflammatory joint disease. Histopathology 1999; 34: 144–53.
3. Dunn AL. Pathophysiology, diagnosis and prevention of arthropathy in patients with haemophilia. Haemophilia, 2011; 17: 571– 8.
4. Stephensen D. Rehabilitation of patients with haemophilia after orthopaedic surgery: a case study. Haemophilia, 2005; 11(Suppl. 1): 26-29.
5. Iorio A, Marchesini E, Marcucci M, Stobart K, Chan AFC. Clotting Factor Concentrates Given to Prevent Bleeding and Bleeding-Related Complications in People with Haemophilia A or B (Review). Cochrane Libr 2011 (Issue 9). Chichester: Wiley, 2011.
6. Iorio A, Matino D, D'Amico R, Makris M. Recombinant Factor VIIa Concentrate Versus Plasma Derived Concentrates for the Treatment of Acute Bleeding Episodes in People with Haemophilia and Inhibitors (Review). Cochrane Database of Systematic Reviews (issue 8). Chichester: Wiley, 2010.
7. Feldman B, Pai M, Rivard G et al. Tailored prophylaxis in severe haemophilia A: interim results from the first 5 years of the Canadian Haemophilia Primary Prophylaxis Study. Journal of Thrombosis and Haemostasis 2006;4: 1228–36.
8. Manco-Johnson M, Abshire T, Shapiro A et al. Prophylaxis versus episodic treatment to prevent joint disease in boys with severe haemophilia. The New England Journal of Medicine 2007; 357: 535–44.
9. Pergantou H, Platokouki H, Matsinos G et al. Assessment of the progression of haemophilic arthropathy in children. Haemophilia, 2010; 16: 124-129.
10. Stephensen D, Drechsler W, Scott O. Comparison of muscle strength and in-vivo muscle morphology in young children with haemophilia and those of age-matched peers. Haemophilia 2012; 18: e302–10.
11. Falk B, Portal S, Tiktinsky R, et al. Anaerobic power and muscle strength in young hemophilia patients. Medicine Science Sports and Exercise 2000; 32: 52–7.
12. Falk B, Portal S, Tiktinsky R et al. Bone properties and muscle strength of young haemophilia patients. Haemophilia 2005; 11: 380-6.
13. Stephensen D, Drechsler Wl & Scott OM. Influence of ankle plantar flexor muscle architecture and strength on gait in boys with haemophilia in comparison to typically developing children. Haemophilia, 2014; 20(3): 413-420.

For peer review only

For peer review only - http://bmjopen.bmj.com/site/about/guidelines.xhtml

14. Stephensen D, Taylor S, Bladen M, Drechsler W. Relationship between physical function and biomechanical gait patterns in boys with haemophilia. Haemophilia. 2016;22(6):e512-e518.

15. Geneen LJ, Moore RA, Clarke C, Martin D, Colvin LA, Smith BH.Physical activity and exercise for chronic pain in adults: an overview of Cochrane Reviews. Cochrane Database Syst Rev. 2017; 24:4:CD011279

16. Blamey G, Forsyth A, Zourikian N, Short L, Jankovic N, De Kleijn P, Flannery T. Comprehensive elements of a physiotherapy exercise programme in haemophilia--a global perspective. Haemophilia. 2010;16 (Suppl 5):136-45.

17. Strike K, Mulder K, Michael R. Exercise for haemophilia. Cochrane Database of Systematic Reviews 2016, Issue 12. Art. No.: CD011180.

18. Eid MA, Ibrahim MM, Aly SM. Effect of resistance and aerobic exercises on bone mineral density, muscle strength and functional ability in children with hemophilia. The Egyptian Journal of Medical Human Genetics (2014) 15, 139–147.

19. Mohamed RA, Sherief AEA. Bicycle ergometer versus treadmill on balance and gait parameters in children with hemophilia. The Egyptian Journal of Medical Human Genetics (2015) 16, 181–187.

20. Chan A-W, Tetzlaff JM, Altman DG, Laupacis A, Gøtzsche PC, Krleža-Jerić K, Hróbjartsson A, Mann H, Dickersin K, Berlin J, Doré C, Parulekar W, Summerskill W, Groves T, Schulz K, Sox H, Rockhold FW, Rennie D, Moher D. SPIRIT 2013 Statement: Defining standard protocol items for clinical trials. Ann Intern Med. 2013;158(3):200-207

21. Hilliard P, Funk S, Zourikian N, Bergstrom BM, Bradley CS, McLimont M, et al. Hemophilia joint health score reliability study. Haemophilia. 2006; 12(5): 518-25.

22. Martinoli C, Della Casa Alberighi O, Di Minno G, et al. Development and definition of a simplified scanning procedure and scoring method for Haemophilia Early Arthropathy Detection with Ultrasound (HEAD-US). Thrombosis and Haemostasis, 2013; 109(6): 1170-1179.

23. Stephensen D, Classey S, Harbidge H, Patel V, Taylor S, Wells A. Physiotherapist inter-rater reliability of the Haemophilia Early Arthropathy Detection with Ultrasound (HEAD-US) protocol. Haemophilia. 2018; 24(3):471-476

24. Wille N et al. (2010). Development of the EQ-5D-Y: a child-friendly version of the EQ-5D. Quality of Life Research; 19(6):887-97.

For peer review only - http://bmjopen.bmj.com/site/about/guidelines.xhtml

Figure Legend

Figure 1. Flowchart of participant journey through the feasibility study

For peer review only

For peer review only - http://bmjopen.bmj.com/site/about/guidelines.xhtml

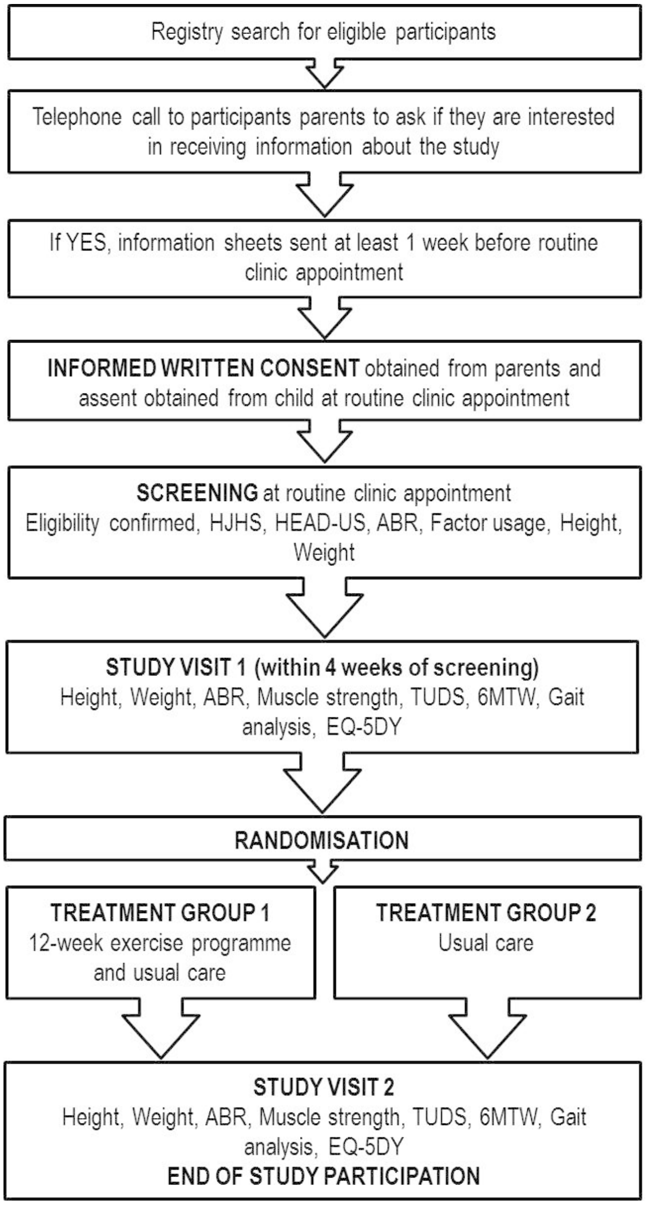

Figure 1. Flowchart of participant journey through the feasibility study

109x205mm (150 x 150 DPI)

Reporting checklist for protocol of a clinical trial.

Based on the SPIRIT guidelines.

Instructions to authors

Complete this checklist by entering the page numbers from your manuscript where readers will find each of the items listed below.

Your article may not currently address all the items on the checklist. Please modify your text to include the missing information. If you are certain that an item does not apply, please write "n/a" and provide a short explanation.

Upload your completed checklist as an extra file when you submit to a journal.

In your methods section, say that you used the SPIRIT reporting guidelines, and cite them as:

Chan A-W, Tetzlaff JM, Altman DG, Laupacis A, Gøtzsche PC, Krleža-Jerić K, Hróbjartsson A, Mann H, Dickersin K, Berlin J, Doré C, Parulekar W, Summerskill W, Groves T, Schulz K, Sox H, Rockhold FW, Rennie D, Moher D. SPIRIT 2013 Statement: Defining standard protocol items for clinical trials. Ann Intern Med. 2013;158(3):200-207

		Reporting Item	Page Number
Title	#1	**Descriptive title identifying the study design, population, interventions, and, if applicable, trial acronym**	1
Trial registration	#2a	**Trial identifier and registry name. If not yet registered, name of intended registry**	N/A Feasibility study
Trial registration: data set	#2b	**All items from the World Health Organization Trial Registration Data Set**	N/A
Protocol version	#3	**Date and version identifier**	5
Funding	#4	**Sources and types of financial, material, and other support**	12
Roles and responsibilities: contributorship	#5a	**Names, affiliations, and roles of protocol contributors**	12

For peer review only

Roles and responsibilities: sponsor contact information	#5b	**Name and contact information for the trial sponsor**	11
Roles and responsibilities: sponsor and funder	#5c	**Role of study sponsor and funders, if any, in study design; collection, management, analysis, and interpretation of data; writing of the report; and the decision to submit the report for publication, including whether they will have ultimate authority over any of these activities**	11
Roles and responsibilities: committees	#5d	**Composition, roles, and responsibilities of the coordinating centre, steering committee, endpoint adjudication committee, data management team, and other individuals or groups overseeing the trial, if applicable (see Item 21a for data monitoring committee)**	11
Background and rationale	#6a	**Description of research question and justification for undertaking the trial, including summary of relevant studies (published and unpublished) examining benefits and harms for each intervention**	4-5
Background and rationale: choice of comparators	#6b	**Explanation for choice of comparators**	6-8
Objectives	#7	**Specific objectives or hypotheses**	5
Trial design	#8	**Description of trial design including type of trial (eg, parallel group, crossover, factorial, single group), allocation ratio, and framework (eg, superiority, equivalence, non-inferiority, exploratory)**	6-8
Study setting	#9	**Description of study settings (eg, community clinic, academic hospital) and list of countries where data will be collected. Reference to where list of study sites can be obtained**	6-8

For peer review only

Eligibility criteria	#10	**Inclusion and exclusion criteria for participants. If applicable, eligibility criteria for study centres and individuals who will perform the interventions (eg, surgeons, psychotherapists)**	5-6
Interventions: description	#11a	**Interventions for each group with sufficient detail to allow replication, including how and when they will be administered**	7-8
Interventions: modifications	#11b	**Criteria for discontinuing or modifying allocated interventions for a given trial participant (eg, drug dose change in response to harms, participant request, or improving / worsening disease)**	8,11
Interventions: adherance	#11c	**Strategies to improve adherence to intervention protocols, and any procedures for monitoring adherence (eg, drug tablet return; laboratory tests)**	7-8
Interventions: concomitant care	#11d	**Relevant concomitant care and interventions that are permitted or prohibited during the trial**	7-8
Outcomes	#12	**Primary, secondary, and other outcomes, including the specific measurement variable (eg, systolic blood pressure), analysis metric (eg, change from baseline, final value, time to event), method of aggregation (eg, median, proportion), and time point for each outcome. Explanation of the clinical relevance of chosen efficacy and harm outcomes is strongly recommended**	8-10
Participant timeline	#13	**Time schedule of enrolment, interventions (including any run-ins and washouts), assessments, and visits for participants. A schematic diagram is highly recommended (see Figure)**	Figure 1
Sample size	#14	**Estimated number of participants needed to achieve study objectives and how it was determined, including clinical and statistical**	5

For peer review only

		**assumptions supporting any sample size calculations**	
Recruitment	#15	**Strategies for achieving adequate participant enrolment to reach target sample size**	5-6
Allocation: sequence generation	#16a	**Method of generating the allocation sequence (eg, computer-generated random numbers), and list of any factors for stratification. To reduce predictability of a random sequence, details of any planned restriction (eg, blocking) should be provided in a separate document that is unavailable to those who enrol participants or assign interventions**	6
Allocation concealment mechanism	#16b	**Mechanism of implementing the allocation sequence (eg, central telephone; sequentially numbered, opaque, sealed envelopes), describing any steps to conceal the sequence until interventions are assigned**	6
Allocation: implementation	#16c	**Who will generate the allocation sequence, who will enrol participants, and who will assign participants to interventions**	6
Blinding (masking)	#17a	**Who will be blinded after assignment to interventions (eg, trial participants, care providers, outcome assessors, data analysts), and how**	6-7
Blinding (masking): emergency unblinding	#17b	**If blinded, circumstances under which unblinding is permissible, and procedure for revealing a participant's allocated intervention during the trial**	N/A Clinical care team aware of allocation Page 6
Data collection plan	#18a	**Plans for assessment and collection of outcome, baseline, and other trial data, including any related processes to promote data quality (eg, duplicate measurements, training of assessors) and a description of study instruments (eg, questionnaires, laboratory tests) along with their reliability and validity, if known. Reference to**	8-10

For peer review only

		where data collection forms can be found, if not in the protocol	
Data collection plan: retention	#18b	Plans to promote participant retention and complete follow-up, including list of any outcome data to be collected for participants who discontinue or deviate from intervention protocols	7
Data management	#19	Plans for data entry, coding, security, and storage, including any related processes to promote data quality (eg, double data entry; range checks for data values). Reference to where details of data management procedures can be found, if not in the protocol	10
Statistics: outcomes	#20a	Statistical methods for analysing primary and secondary outcomes. Reference to where other details of the statistical analysis plan can be found, if not in the protocol	10
Statistics: additional analyses	#20b	Methods for any additional analyses (eg, subgroup and adjusted analyses)	10
Statistics: analysis population and missing data	#20c	Definition of analysis population relating to protocol non-adherence (eg, as randomised analysis), and any statistical methods to handle missing data (eg, multiple imputation)	10
Data monitoring: formal committee	#21a	Composition of data monitoring committee (DMC); summary of its role and reporting structure; statement of whether it is independent from the sponsor and competing interests; and reference to where further details about its charter can be found, if not in the protocol. Alternatively, an explanation of why a DMC is not needed	11
Data monitoring: interim analysis	#21b	Description of any interim analyses and stopping guidelines, including who will have access to these interim results and make the final decision to terminate the trial	11

Harms	#22	**Plans for collecting, assessing, reporting, and managing solicited and spontaneously reported adverse events and other unintended effects of trial interventions or trial conduct**	11
Auditing	#23	**Frequency and procedures for auditing trial conduct, if any, and whether the process will be independent from investigators and the sponsor**	11
Research ethics approval	#24	**Plans for seeking research ethics committee / institutional review board (REC / IRB) approval**	12
Protocol amendments	#25	**Plans for communicating important protocol modifications (eg, changes to eligibility criteria, outcomes, analyses) to relevant parties (eg, investigators, REC / IRBs, trial participants, trial registries, journals, regulators)**	12
Consent or assent	#26a	**Who will obtain informed consent or assent from potential trial participants or authorised surrogates, and how (see Item 32)**	6
Consent or assent: ancillary studies	#26b	**Additional consent provisions for collection and use of participant data and biological specimens in ancillary studies, if applicable**	N/A
Confidentiality	#27	**How personal information about potential and enrolled participants will be collected, shared, and maintained in order to protect confidentiality before, during, and after the trial**	10
Declaration of interests	#28	**Financial and other competing interests for principal investigators for the overall trial and each study site**	12
Data access	#29	**Statement of who will have access to the final trial dataset, and disclosure of contractual agreements that limit such access for investigators**	-
Ancillary and post trial care	#30	**Provisions, if any, for ancillary and post-trial care, and for compensation to those who suffer harm from trial participation**	-

For peer review only

Dissemination policy: trial results	#31a	**Plans for investigators and sponsor to communicate trial results to participants, healthcare professionals, the public, and other relevant groups (eg, via publication, reporting in results databases, or other data sharing arrangements), including any publication restrictions**	12
Dissemination policy: authorship	#31b	**Authorship eligibility guidelines and any intended use of professional writers**	-
Dissemination policy: reproducible research	#31c	**Plans, if any, for granting public access to the full protocol, participant-level dataset, and statistical code**	-
Informed consent materials	#32	**Model consent form and other related documentation given to participants and authorised surrogates**	-
Biological specimens	#33	**Plans for collection, laboratory evaluation, and storage of biological specimens for genetic or molecular analysis in the current trial and for future use in ancillary studies, if applicable**	N/A

The SPIRIT checklist is distributed under the terms of the Creative Commons Attribution License CC-BY-ND 3.0. This checklist can be completed online using https://www.goodreports.org/, a tool made by the EQUATOR Network in collaboration with Penelope.ai

For peer review only

Protocol for a feasibility randomised controlled trial of a musculoskeletal exercise intervention versus usual care for children with haemophilia

Journal:	*BMJ Open*
Manuscript ID	bmjopen-2019-029474.R1
Article Type:	Protocol
Date Submitted by the Author:	01-May-2019
Complete List of Authors:	Hashem, Ferhana; University of Kent, Centre for Health Service Studies<br>Bladen, Melanie; Great Ormond Street Hospital For Children NHS Foundation Trust, Haemophilia Centre<br>Carroll, Liz; Haemophilia Society<br>Dodd, Charlene; East Kent Hospitals University NHS Foundation Trust, Haemophilia Centre<br>Drechsler, Wendy; King's College London, School of Population Health & Environmental Sciences<br>Lowery, David; University of Kent, Centre for Health Service Studies<br>Patel, Vishal; Barts Health NHS Trust, Haemophilia Centre<br>Pellatt-Higgins, T; University of Kent, Centre for Health Services studies<br>Saloniki, Eirini; University of Kent, Centre for Health Service Studies<br>Stephensen, David; East Kent Hospitals University NHS Foundation Trust, Haemophilia Centre; Barts Health NHS Trust, Haemophilia Centre
**Primary Subject Heading**:	Haematology (incl blood transfusion)
Secondary Subject Heading:	Paediatrics, Rehabilitation medicine, Sports and exercise medicine
Keywords:	haemophilia, physiotherapy, exercise, muscle strength, physical function

Note: The following files were submitted by the author for peer review, but cannot be converted to PDF. You must view these files (e.g. movies) online.

DOLPHIN Feasibility RCT - SPIRIT_checklist.docx

SCHOLARONE™ Manuscripts

Protocol for a feasibility randomised controlled trial of a musculoskeletal exercise intervention versus usual care for children with haemophilia

Hashem F[1],Bladen M[2], Carroll L[3], Dodd C[5], Drechsler WI[4], Lowery D[1], Patel V[6], Pellatt-Higgins T[1], Saloniki E-C[1,7], Stephensen D[5,6]

[1]University of Kent, Centre for Health Service Studies, Canterbury, United Kingdom

[2]Great Ormond Street Hospital For Children NHS Foundation Trust, Haemophilia Centre, London, United Kingdom

Institute of Child Health, University College London, London, United Kingdom

[3]Haemophilia Society UK, London, United Kingdom

[4]Kings College London, School of Population Health & Environmental Sciences, London, United Kingdom

[5]East Kent Hospitals University NHS Trust, Haemophilia and Thrombosis Centre, Canterbury, United Kingdom

[6]Royal London Hospital, Haemophilia Centre, London, United Kingdom

[7]University of Kent, Personal Social Services Research Unit, Canterbury, United Kingdom

Corresponding Author: Dr David Stephensen, East Kent Hospitals University NHS Trust, Haemophilia and Thrombosis Centre, Canterbury, United Kingdom. david.stephensen@nhs.net

Key Words: Haemophilia, Physiotherapy, Exercise, Muscle strength, Physical function

Abstract word count: 277 words

Manuscript word count: 3283 words (excluding Figure and References)

For peer review only - http://bmjopen.bmj.com/site/about/guidelines.xhtml

ABSTRACT

Introduction: Haemophilia is a rare, inherited disorder in which blood does not clot normally, resulting in bleeding into joints and muscles. Long-term consequence is disabling joint pain, stiffness, muscle weakness, atrophy and reduced mobility. The purpose of this proposed feasibility of a randomised controlled trial (RCT) is to test the feasibility of an age-appropriate physiotherapy intervention designed to improve muscle strength, posture and the way boys use their joints during walking and everyday activities.

Methods and analysis: A small-scale two-centre RCT of a 12-week muscle strengthening exercise intervention versus usual care for young children with haemophilia will be conducted. Primary outcomes will be safety and adherence to the exercise intervention. Secondary outcomes will include: recruitment, retention and adverse event rates, clinical data, muscle strength, joint biomechanics and foot loading patterns during walking, six-minute timed walk, timed-up-and-down-stairs, EQ-5D-Y, participants' perceptions of the study, training requirements and relevant costs. Recruitment, follow-up, safety and adherence rates will be described as percentages. Participant diary and interview data will be analysed using a framework analysis. Demographic and disease variable distributions will be analysed for descriptive purposes and co-variant analysis. Estimates of differences between treatment arms (adjusted for baseline), and 75% and 95% confidence intervals will be calculated.

Ethics and Dissemination: The study has ethical approval from the London - Fulham Research Ethics Committee (17/LO/2043) as well as Health Research Authority approval. As well as informing the design of the definitive trial, results of this study will be presented at local, national and international physiotherapy and haemophilia meetings as well as manuscripts submitted to peer-reviewed journals. We will also share the main findings of the study to all participants and the Haemophilia Society.

For peer review only

ARTICLE SUMMARY

Strengths and limitations of this study

- This study focuses on the feasibility of a planned multi-centre randomised controlled trial of a muscle strengthening exercise intervention versus usual care in children with haemophilia
- It is impossible to blind those in receipt and those delivering the intervention as to the group allocation, but we will blind the assessor as to group allocation
- The study will determine whether recruitment and adherence strategies are appropriate, and whether the exercises are safe and can be completed by children with haemophilia
- At the end of this study we will not be able to answer the question "Does exercises help improve the long term health of children with haemophilia?", but we will know whether such a study could be undertaken and the best way to deliver the intervention

For peer review only - http://bmjopen.bmj.com/site/about/guidelines.xhtml

INTRODUCTION

Haemophilia is a rare, inherited disorder in which blood does not clot normally. Repeated spontaneous and traumatic bleeding into joints and muscles is the result; the ankle joint being the most common.[1] Long-term consequence is chronic arthropathy, a pattern of disabling joint pain, stiffness, muscle weakness, atrophy and reduced mobility.[2-4] The introduction of prophylaxis with clotting factor concentrates has reduced the number of bleeding episodes experienced by children, changing their lifestyle and long term outlook.[5-6] However, evidence indicates that despite prophylaxis, boys still bleed 1-2 times per year, with relatively few bleeding episodes being required to initiate bone destruction, and in some cases, articular erosion is evident with no history of joint bleeding.[1,7-9] Furthermore, children are more susceptible to blood induced damage than adults and, once established is progressive and irreversible.[10]

It is well established that muscle weakness is associated with haemophilic arthropathy in adults[4] and it is now becoming apparent that muscle strength is reduced in children, prior to the onset of clinical arthropathy.[11] In a study of 10 adolescent and adult haemophiliacs with a history of unilateral knee bleeding, Falk *et al.* (2005) found that strength of the knee extensors and flexors in haemophilic children were significantly weaker than those of the age-matched controls.[12-13] More recently, Stephensen *et al.* (2012) observed that despite normal clinical examinations and comparable levels of physical activity, young haemophilic boys with a history of ankle joint haemorrhage had significantly reduced dynamic muscle strength together with atrophy of the knee extensors, ankle dorsi- and plantarflexors when compared to age and size-matched typically developing peers.[11] Furthermore, muscle weakness of the leg is strongly correlated to muscle atrophy, reduced walking distances, slower ascent and descent of stairs, as well as reduced ankle joint motion, greater knee flexion motion and forces at the knee and ankle joint during walking.[14-15]

Therapeutic exercise is an important component of the management of other forms of arthropathy (e.g. osteoarthritis, rheumatoid arthritis) [16] and it would appear logical that exercise would be effective for people with haemophilia. There is growing evidence of the benefits of incorporating muscle strengthening exercise in their management of arthropathy.[17] It is the view of clinicians that increases in muscular strength and postural control might improve motor performance and cardiovascular fitness, limit exaggerated end-range joint movement, and

For peer review only - http://bmjopen.bmj.com/site/about/guidelines.xhtml

promote optimal transfer of weight-bearing forces through joints thereby minimising muscle imbalance, synovial impingement and associated haemarthroses or synovitis.[18] However, there is a lack of evidence to support these assumptions. A recent Cochrane Review evaluating the safety and effectiveness of exercise for people with haemophilia reported four randomly controlled studies of an exercise intervention in children with the condition and concluded the studies were of low or very low quality, due to small sample sizes and potential bias.[17] Furthermore, no paediatric study compared a muscle strengthening intervention to a control group or intervention without muscle strengthening exercises. Several studies compared two multi-component exercise interventions that included broad muscle strengthening as well as treadmill walking and cycle ergometry and it is not possible to determine the effect of muscle strengthening on muscle strength or its effect on physical function.[19-20] Furthermore, pre-adolescent and adolescent boys aged between 10 and 14 were included in these studies, and it is not known if the groups were matched for pubertal status.

Despite the apparent benefit, there is a lack of robust evidence to determine whether muscle strengthening exercise can improve or negatively affect outcomes for young children with haemophilia. The purpose of this proposed feasibility RCT is to test the feasibility of an age-appropriate physiotherapy intervention designed to improve muscle strength, posture and the way boys use their joints during walking and everyday activities.

METHODS AND ANALYSIS

The protocol version for this feasibility study is Version 2 – 19/12/2017. The Standard Protocol Items: Recommendations for Interventional Trials (SPIRIT) guidelines[21] have been used to report the study protocol.

<u>Participants</u>

Participants will be invited from two haemophilia centres. Twenty boys with severe or moderate haemophilia A or B with or without inhibitors on prophylactic treatment with coagulation factor concentrate, aged 6-11 years will be recruited. Exclusion criteria are: past history of fracture or trauma to the lower limb; orthopaedic surgery; acquired brain injury or any other disturbance of the central nervous system; joint or muscle bleed in the lower limb in the past 6 weeks; presence of lower limb pain or unable to fully comply with verbal instructions.

For peer review only - http://bmjopen.bmj.com/site/about/guidelines.xhtml

Participants will be identified from each Haemophilia Centre's database of registered patients. The parents or guardians of eligible participants will be approached initially by the principle investigator at each site via telephone to provide a brief verbal explanation of the research study and ask if they would be happy to receive a patient information sheet. Should they be interested in participating in the study they will receive, by post, the patient information sheet (one for parents and a separate age appropriate version for children) at least one week prior to their next clinic appointment. This will allow for a 'cooling-off' period of a minimum of one week for the potential participants. The researcher will then be available for questions by telephone or at the participant's next clinic appointment.

At their clinic appointment, the investigator will meet with the participant and parent/ legal guardian to complete the consent form as appropriate. The investigator will further explain the study procedures, why they are being asked to consider including their child in this study and the associated risks and benefits of being included. They will then be given an opportunity to ask any further questions. The child/ parent/ legal guardian will then be asked to confirm if the potential participant agrees to take part in the study. If consent is agreed a parental consent form will be completed by the parent/ guardian and a copy will be provided. If at any point during the study process the parent/guardian does not consent to inclusion in the study then any data collected will be destroyed. Any decision to refuse or withdraw from the study will in no way influence the services that they receive. Once the child's parent/guardian has given consent, the child must also indicate that they do not object to participating in the research activity. This participant 'assent' will be sought.

Study design

Following written informed consent and screening of eligibility criteria, randomisation of eligible participants for the study will be performed with the online randomisation service http://sealedenvelope.com/. Utilising the secure, centralised and independent service, participants will be randomly allocated into one of two groups (Treatment Group 1 will receive a 12-week muscle strengthening exercise intervention; Treatment Group 2 will receive usual physiotherapy care for 12 weeks) on a ratio of 1:1. Allocation will be stratified by centre to ensure the same numbers of participants at each site are allocated to the two groups. The un-blinded principal investigator at each site will be issued with a password to access the service to create a new randomisation for the study participants. Those allocated to Treatment Group 2

For peer review only

For peer review only - http://bmjopen.bmj.com/site/about/guidelines.xhtml

will be offered the intervention after their end of study follow-up assessment. Data will be collected prior to and immediately post intervention (Figure 1).

It is impossible to blind those in receipt and those delivering the intervention as to the group allocation. However, it will be possible – although perhaps challenging - to blind the assessor as to group allocation. Consequently, we will attempt a single blind approach in which the participant and physiotherapists delivering the intervention will be encouraged to withhold their group allocation from the assessors collecting data. We will use a range of strategies, including: instruction in the participant literature; intervention protocol and with frequent verbal prompts. In order to assess the efficacy of blinding, we will ask assessors to record which group they think participants have been allocated to at the end of each assessment. This will be presented as a binary forced choice. There are no circumstances under which it would be necessary to un-blind assessors.

<u>Intervention</u>

The intervention, developed by expert clinicians and patients utilising a modified nominal group technique is a 24-session, 12-week programme designed as 2-week progressive levels (intensity and or load) with no more than ten exercises in each session, taking 25-30 minutes to complete (Figure 2). The intervention aims to master movement control and emphasizes body-weight strength development. Pictures of the exercises and instructions for each phase will be provided in 2-week exercise diaries. Participants will be asked to complete the exercises twice per week; once with the physiotherapist who will visit the participant at home, and once supervised by their parents/ guardian. Completion of exercises, along with treatment regime, any adverse events and comments in relation to the exercise programme will be recorded in the exercise diary. They will also record participation in physical activities, including type, time and intensity. According to predetermined criteria (safe and competent), the physiotherapist will determine if it is appropriate for the participant to progress to the next exercise level. On return of the exercise diary every 2 weeks, participants will be provided with a gift voucher.

<u>Usual care</u>

Participants will continue to receive their usual care as recommended and prescribed by the haematologist, physiotherapist and other members of the healthcare team. This will include factor concentrate treatment, any physiotherapy or surgical intervention or advice that is recommended by the clinical care team. There are currently no standardised treatment

For peer review only - http://bmjopen.bmj.com/site/about/guidelines.xhtml

recommendations for children without apparent joint damage. The researchers will not make or initiate any changes to routine treatment. Any change to or initiation of new treatment or intervention will be recorded and the treating clinician will determine whether it is appropriate for the participant to continue in the study. Participants will be provided with a diary to record participation in physical activities, including type, time and intensity. Participants will be telephoned weekly to encourage dairy completion. On return of the diary every 2 weeks, participants will be provided with a gift voucher.

Outcome measures

The primary outcome measures are:

- Safety will be evaluated using annualised bleeding rates (ABR), coagulation factor usage (what the patient actually administers and records on their routine electronic diary) and reported adverse events and reactions associated with the intervention
- Adherence to the exercise intervention will be evaluated by the number of exercise session completed as a percentage of the prescribed exercise sessions. Data will be extracted from participant diaries

Secondary outcome measures are:

- Recruitment rate determined as the participants who volunteer to participate in the study as a percentage of those that are invited/ eligible
- Willingness to be randomised; evaluated at the end of study through semi-structured interviews that will be audio-recorded and be transcribed by a member of the research team
- Participant's acceptability of the trial and intervention: using semi-structured interviews, conducted at the completion of the study, the participants will be asked their views on acceptability of the trial and intervention, recruitment and exercise adherence. The interview will be audio-recorded and be transcribed by a member of the research team
- Training required to deliver the intervention and collect outcome data at study visits: using semi-structured interviews, conducted at the completion of the study, the physiotherapists will be asked their views on acceptability of the trial and intervention, recruitment and exercise adherence. They will also be asked about the training requirements for delivering the intervention as well as the appropriateness of the outcomes piloted in terms of training, length of time to complete and clinical value of the data. The interview will be audio-recorded and be transcribed by a member of the research team

For peer review only - http://bmjopen.bmj.com/site/about/guidelines.xhtml

- Haemophilia Joint Health Score (HJHS) – a clinical examination of swelling, muscle atrophy, strength, joint crepitus, range of motion and pain at the ankle, knee and elbow joints. The HJHS is a standardised multi-item assessment, currently used in clinic as part of routine care to record musculoskeletal health[22]
- Haemophilic synovitis and arthropathy – The presence and severity of signs of haemophilic synovitis and arthropathy will be evaluated using the Haemophilia Early Arthropathy Detection with Ultrasound (HEAD-US) score. The HEAD-US protocol is a standardised scanning protocol, used to identify the presence and severity of synovitis, articular cartilage and chondral bone defects in the ankle, knee and elbow joint. The scanning procedure takes approximately 2-3 minutes per joint and we have recently shown good inter-rater repeatability of this protocol when performed by physiotherapists[23-24]
- Muscle strength of the knee extensors and ankle dorsi- and plantarflexors – Maximum torque will be recorded with a hand held dynamometer (Manual Muscle Tester, Lafayette Instrument, USA) using our standardised protocol with excellent test-retest repeatability[15]
- Joint biomechanics and motion during walking – Temporal spatial and three-dimensional (3D) joint motion during gait will be recorded using an inertial magnetic measurement gait analysis system (MVN BIOMECH Awinda, Xsens Technologies, Netherlands). Sensor units will be attached to the feet, lower legs, thighs, and pelvis with elastic straps. Motion of the sensors will be used to calculate temporal spatial and 3D lower limb joint motion
- Static and dynamic foot loading patterns – will be evaluated with a plantar pressure platform (HR Mat, Tekscan, USA). Centre of pressure motion in respect of distance and direction travelled, together with contact time as a measure of foot symmetry of foot loading for various foot segments (heel, midfoot and forefoot) along with distribution of peak pressure and pressure-time integrals will be evaluated
- Six-minute timed walk (6MTW) – Distance walked in 6 minutes. Two marker cones will be placed 30 metres apart along a corridor and each subject will be asked to walk back and forth between the cones as fast as they can for 6 minutes.[15]
- Timed up and down stairs (TUDS) – Time taken to ascend and descend a flight of 12 steps. The children will stand 30-centremetres from the bottom step and asked to walk quickly but safely up the stairs, turn around on the top step and come all the way down until both feet land on the bottom step. They will be instructed to step on every step, use the hand rail if they want and not to run.[15]

For peer review only - http://bmjopen.bmj.com/site/about/guidelines.xhtml

- EQ-5D-Y – a five dimension (mobility, self-care, usual activities, pain/discomfort, anxiety/depression) questionnaire measuring health-related quality of life.[25]
- Costs relevant to the study – intervention costs, health and social care costs, out-of-pocket expenses and time off work costs. Healthcare costs (using National Health Service reference costs were possible) will include; training and delivering the intervention, equipment, overhead costs, visits to hospital/ haemophilia clinic, general practitioner, other healthcare services, investigations, hospitalisation, treatment, coagulation clotting factor concentrate and medication. Patient and family costs will include travel and time off work. Feasibility of collecting these data will be explored along with the resource consequences.

Data will be collected at the respective Haemophilia centres. All data collected related to the study will be confidential and anonymous. The participant will be allocated a randomised participant number code and all data collected will relate to this code. A record of which code belongs to which subject will be stored in a locked cabinet separate from the study data. Only the principle investigator (who will be a member of the participant's direct care team) will have access to this documentation. The direct care team and the principal investigator will have access to participant's personal and identifiable information during the study. Consent will be sought by the lead researcher for the research team to have access to this personal information.

Analysis methods

Recruitment, follow-up, safety and adherence rates will be described as percentages. Participant diary and interview data will be analysed to identify factors that influence these rates and acceptability of the trial using a framework analysis. Interview data from physiotherapists will be analysed using a framework analysis to determine training required to deliver the intervention and collect outcome data. Feasibility of collecting healthcare cost data will be explored along with the resource consequences. Costs will be analysed and reported using descriptive statistics. Demographic and disease variable distributions will be analysed for descriptive purposes and co-variant analysis. Walking variables will be normalised to leg length and muscle strength to body mass. Group mean and standard deviation (median and inter-quartile range where appropriate) will be calculated and reported. Change from baseline data will be compared between groups at all study time points using analysis of covariance with baseline as a covariate and a factor for centre. Estimates of differences between treatment arms in the changes from baseline (adjusted for baseline), and 75% and 95% confidence

For peer review only - http://bmjopen.bmj.com/site/about/guidelines.xhtml

intervals will be calculated. Group mean and standard deviation (median and inter-quartile range where appropriate) will be calculated pre and post intervention. These data will allow us to estimate the potential effect of the intervention and provide estimates of variability to inform the sample size required for a full RCT, as well as to refine the outcome measures by evaluating their responsiveness to changes in musculoskeletal health.

For peer review only

For peer review only - http://bmjopen.bmj.com/site/about/guidelines.xhtml

Patient and Public Involvement

Ten specialist haemophilia physiotherapists in the United Kingdom plus those experienced in sports injury were invited to take part in a Nominal Group Technique (NGT) consensus discussion to share their experience and expertise to contribute to the development of the exercise intervention. Patients' priorities, experience, and preferences informed the study design, recruitment as well as the design and delivery of the intervention. The exercise programme was demonstrated to five boys with haemophilia and their parents who were members of the Haemophilia Society, national UK patient organisation. The parents and children were asked about what they thought about the exercises and whether they could undertake them on a regular basis, where they thought the best place was for undertaking them, and how they would like to receive the information on the exercise programme. Parents and children were then asked questions about how they would feel about taking part in study testing the benefits of the exercises, issues around being allocated randomly into study groups, and then questions about what would encourage the children to continue on the exercise programme with discussions focusing upon types of rewards and incentivisation.

Management and oversight of the study

East Kent Hospital University Foundation Trust is the study sponsor. They have overall responsibility for governance of the study and have no direct role in study design, data collection, management, analysis, interpretation of data and reports and publications that arise from the study.

A Research Monitoring Committee (RMC) has been established and will meet six-monthly after the study has commenced. The RMC will be responsible for monitoring milestones and targets, data, safety, recruitment, reviewing and interpreting the results. The RMC will be chaired by an independent chair from outside the research team group. Additional meetings may be called to review any serious adverse events (SAE), adverse events (AE) and adverse reactions (AR). The Chief Investigator will: co-ordinate day to day running of the research; complete ethical approvals; communicate with recruitment sites; complete training of physiotherapists to deliver the intervention; undertake research initiation and monitoring site visits; co-ordinate data analysis and interpretation. The Chief Investigator will notify the RMC in the case of AEs and ARs. The Principal Investigator at each site will be the physiotherapist delivering the intervention. They will be responsible for recruitment and screening participants according to the inclusion and exclusion criteria, reporting AEs and ARs and local governance requirements.

For peer review only - http://bmjopen.bmj.com/site/about/guidelines.xhtml

ETHICS AND DISSEMINATION

Favourable ethical approval was obtained from the Health Research Authority and London - Fulham Research Ethics Committee and (17/LO/2043).

Study newsletters will be published by the RMC and available on the website of the Haemophilia Society. The results of this feasibility study will inform the design of a definitive RCT to test whether muscle strengthening can benefit musculoskeletal health in children with haemophilia, including whether such a study can be achieved and the best way of completing it. The results of the feasibility study will be presented at local, national and international physiotherapy and haemophilia meetings as well as manuscripts submitted to peer-reviewed journals. The main findings of the study will be shared to all participants and the Haemophilia Society.

AUTHORS CONTRIBUTION

DS, MB and WD contributed to the inception of the study. FH, MB, LC, CD, WD, DL, VP, TPH, ES and DS contributed to the study design and planning of the protocol. FH, DS, MB, WD, CD and VP contributed to the design of the intervention. FH and DS drafted the manuscript and FH, MB, LC, CD, WD, DL, VP, TPH, ES and DS critically revised and approved the manuscript.

ACKNOWLEDGEMENTS

The authors would like to acknowledge the contribution of the academic experts and specialist physiotherapists (Wendy Drechsler, Melanie Bladen, Danny Armitage, Trupti Bhandari, Lisa Geuran, Hannah Harbidge, Nicola Hubert, Ann McCarthy, Julie Sparrow, Linda Walsh), the children and parents of the Haemophilia Society for sharing their priorities, experience, and preferences that informed the study design, recruitment as well as the design and delivery of the intervention.

FUNDING:

We gratefully acknowledge the National Institute for Health Research – Research for Patient Benefit Programme for providing funds to support this project (grant number PB-PG-0215-36091).

For peer review only - http://bmjopen.bmj.com/site/about/guidelines.xhtml

CONFLICT OF INTEREST

The authors declare no conflicts of interest.

For peer review only

For peer review only - http://bmjopen.bmj.com/site/about/guidelines.xhtml

REFERENCES

1. Stephensen D, Tait R, Brodie N et al. Changing patterns of bleeding in patients with severe haemophilia A. Haemophilia 2009; 15: 1210–4.
2. Roosendaal G, van Rinsum AC, Vianen ME, et al. Haemophilic arthropathy resembles degenerative rather than inflammatory joint disease. Histopathology 1999; 34: 144–53.
3. Dunn AL. Pathophysiology, diagnosis and prevention of arthropathy in patients with haemophilia. Haemophilia, 2011; 17: 571– 8.
4. Stephensen D. Rehabilitation of patients with haemophilia after orthopaedic surgery: a case study. Haemophilia, 2005; 11(Suppl. 1): 26-29.
5. Iorio A, Marchesini E, Marcucci M, Stobart K, Chan AFC. Clotting Factor Concentrates Given to Prevent Bleeding and Bleeding-Related Complications in People with Haemophilia A or B (Review). Cochrane Libr 2011 (Issue 9). Chichester: Wiley, 2011.
6. Iorio A, Matino D, D'Amico R, Makris M. Recombinant Factor VIIa Concentrate Versus Plasma Derived Concentrates for the Treatment of Acute Bleeding Episodes in People with Haemophilia and Inhibitors (Review). Cochrane Database of Systematic Reviews (issue 8). Chichester: Wiley, 2010.
7. Feldman B, Pai M, Rivard G et al. Tailored prophylaxis in severe haemophilia A: interim results from the first 5 years of the Canadian Haemophilia Primary Prophylaxis Study. Journal of Thrombosis and Haemostasis 2006;4: 1228–36.
8. Manco-Johnson M, Abshire T, Shapiro A et al. Prophylaxis versus episodic treatment to prevent joint disease in boys with severe haemophilia. The New England Journal of Medicine 2007; 357: 535–44.
9. Scott MJ, Xiang H, Hart DP, Palmer B, Collins PW, Stephensen D, Sima CS, Hay CRM. Treatment regimens and outcomes in severe and moderate haemophilia A in the UK: The Thunder Study. Haemophilia, 2019; 25(2):205-212.
10. Pergantou H, Platokouki H, Matsinos G et al. Assessment of the progression of haemophilic arthropathy in children. Haemophilia, 2010; 16: 124-129.
11. Stephensen D, Drechsler W, Scott O. Comparison of muscle strength and in-vivo muscle morphology in young children with haemophilia and those of age-matched peers. Haemophilia 2012; 18: e302–10.
12. Falk B, Portal S, Tiktinsky R, et al. Anaerobic power and muscle strength in young hemophilia patients. Medicine Science Sports and Exercise 2000; 32: 52–7.
13. Falk B, Portal S, Tiktinsky R et al. Bone properties and muscle strength of young haemophilia patients. Haemophilia 2005; 11: 380-6.

For peer review only - http://bmjopen.bmj.com/site/about/guidelines.xhtml

14. Stephensen D, Drechsler WI & Scott OM. Influence of ankle plantar flexor muscle architecture and strength on gait in boys with haemophilia in comparison to typically developing children. Haemophilia, 2014; 20(3): 413-420.

15. Stephensen D, Taylor S, Bladen M, Drechsler W. Relationship between physical function and biomechanical gait patterns in boys with haemophilia. Haemophilia. 2016;22(6):e512-e518.

16. Geneen LJ, Moore RA, Clarke C, Martin D, Colvin LA, Smith BH.Physical activity and exercise for chronic pain in adults: an overview of Cochrane Reviews. Cochrane Database Syst Rev. 2017; 24:4:CD011279

17. Strike K, Mulder K, Michael R. Exercise for haemophilia. Cochrane Database of Systematic Reviews 2016, Issue 12. Art. No.: CD011180.

18. Blamey G, Forsyth A, Zourikian N, Short L, Jankovic N, De Kleijn P, Flannery T. Comprehensive elements of a physiotherapy exercise programme in haemophilia--a global perspective. Haemophilia. 2010;16 (Suppl 5):136-45.

19. Eid MA, Ibrahim MM, Aly SM. Effect of resistance and aerobic exercises on bone mineral density, muscle strength and functional ability in children with hemophilia. The Egyptian Journal of Medical Human Genetics (2014) 15, 139–147.

20. Mohamed RA, Sherief AEA. Bicycle ergometer versus treadmill on balance and gait parameters in children with hemophilia. The Egyptian Journal of Medical Human Genetics (2015) 16, 181–187.

21. Chan A-W, Tetzlaff JM, Altman DG, Laupacis A, Gøtzsche PC, Krleža-Jerić K, Hróbjartsson A, Mann H, Dickersin K, Berlin J, Doré C, Parulekar W, Summerskill W, Groves T, Schulz K, Sox H, Rockhold FW, Rennie D, Moher D. SPIRIT 2013 Statement: Defining standard protocol items for clinical trials. Ann Intern Med. 2013;158(3):200-207

22. Hilliard P, Funk S, Zourikian N, Bergstrom BM, Bradley CS, McLimont M, et al. Hemophilia joint health score reliability study. Haemophilia. 2006; 12(5): 518-25.

23. Martinoli C, Della Casa Alberighi O, Di Minno G, et al. Development and definition of a simplified scanning procedure and scoring method for Haemophilia Early Arthropathy Detection with Ultrasound (HEAD-US). Thrombosis and Haemostasis, 2013; 109(6): 1170-1179.

24. Stephensen D, Classey S, Harbidge H, Patel V, Taylor S, Wells A. Physiotherapist inter-rater reliability of the Haemophilia Early Arthropathy Detection with Ultrasound (HEAD-US) protocol. Haemophilia. 2018; 24(3):471-476

For peer review only - http://bmjopen.bmj.com/site/about/guidelines.xhtml

25. Wille N et al. (2010). Development of the EQ-5D-Y: a child-friendly version of the EQ-5D. Quality of Life Research; 19(6):887-97.

For peer review only

Figure Legend

Figure 1. Flowchart of participant journey through the feasibility study

Figure 2. Description of the exercise intervention

For peer review only

For peer review only - http://bmjopen.bmj.com/site/about/guidelines.xhtml

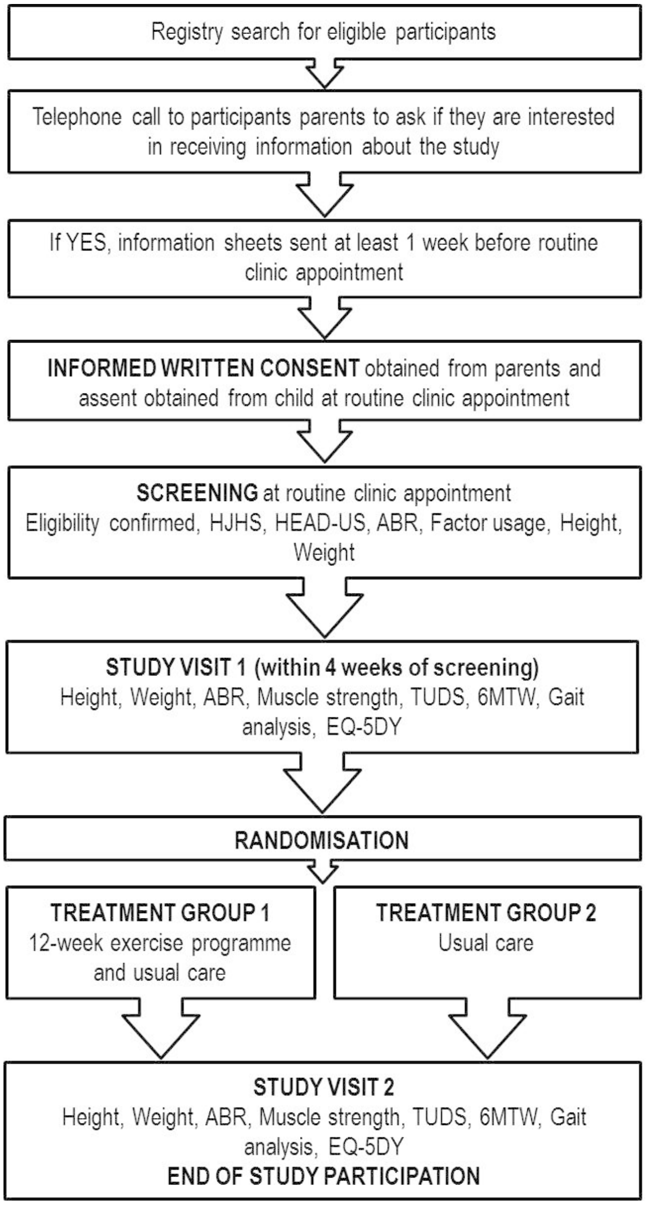

Figure 1. Flowchart of participant journey through the feasibility study

109x205mm (150 x 150 DPI)

	Stretching	Balance	Strengthening	Functional activities
**Week 1-2**	Triangle Stretch<br>Octopus Forward Stretch<br>Turtle Stretch	Tree Pose<br>Football Pose	Assisted double-leg heel raises (2 sets of 5)<br>Foot caterpillar<br>Clamshell<br>Bridge – double leg	Heel toe walk
**Week 3-4**	Triangle Stretch<br>Octopus Forward Stretch<br>Turtle Stretch	Tree pose<br>Football pose (playing hand keepy-up with a balloon)	Double-leg heel raises (2 sets of 5)<br>Foot caterpillar<br>Clamshell<br>Bridge – single leg	Heel toe walk<br>Toe heel walk
**Week 5-6**	Triangle Stretch<br>Octopus Forward Stretch<br>Turtle Stretch	Tree pose on cushion<br>Football pose on cushion	Assisted single leg heel raises (2 sets of 5)<br>Three-legged dog<br>Crab Pose	Toe heel walk<br>Step ups
**Week 7-8**	Triangle Stretch<br>Octopus Forward Stretch<br>Turtle Stretch	Tree pose on cushion<br>Football pose on cushion (playing hand keepy-up with a balloon)	Single-leg heel raises (2 sets of 5)<br>Three-legged dog<br>Forward Warrior Pose	Step ups<br>Crab walk
**Week 9-10**	Triangle Stretch<br>Octopus Forward Stretch<br>Turtle Stretch	Tree pose with eyes closed<br>Scuba diver	Single-leg heel raises (3 sets of 5)<br>Two-legged Dog<br>Forward warrior pose<br>Sideway Warrior Pose	Crab walk<br>Side step ups
**Week 11-12**	Triangle Stretch<br>Octopus Forward Stretch<br>Turtle Stretch	Tree pose on a cushion with eyes closed<br>Scuba diver on cushion	Single-leg heel raises (3 sets of 5)<br>Forward Warrior Pose<br>Sideway Warrior Pose<br>Crab Pose	Step ups<br>Side step ups

Figure 2. Description of exercise intervention

640x361mm (96 x 96 DPI)

Reporting checklist for protocol of a clinical trial.

Based on the SPIRIT guidelines.

Instructions to authors

Complete this checklist by entering the page numbers from your manuscript where readers will find each of the items listed below.

Your article may not currently address all the items on the checklist. Please modify your text to include the missing information. If you are certain that an item does not apply, please write "n/a" and provide a short explanation.

Upload your completed checklist as an extra file when you submit to a journal.

In your methods section, say that you used the SPIRIT reporting guidelines, and cite them as:

Chan A-W, Tetzlaff JM, Altman DG, Laupacis A, Gøtzsche PC, Krleža-Jerić K, Hróbjartsson A, Mann H, Dickersin K, Berlin J, Doré C, Parulekar W, Summerskill W, Groves T, Schulz K, Sox H, Rockhold FW, Rennie D, Moher D. SPIRIT 2013 Statement: Defining standard protocol items for clinical trials. Ann Intern Med. 2013;158(3):200-207

For peer review only

		Reporting Item	Page Number
Title	#1	**Descriptive title identifying the study design, population, interventions, and, if applicable, trial acronym**	1
Trial registration	#2a	**Trial identifier and registry name. If not yet registered, name of intended registry**	N/A Feasibility study
Trial registration: data set	#2b	**All items from the World Health Organization Trial Registration Data Set**	N/A
Protocol version	#3	**Date and version identifier**	5
Funding	#4	**Sources and types of financial, material, and other support**	12
Roles and responsibilities: contributorship	#5a	**Names, affiliations, and roles of protocol contributors**	12
Roles and	#5b	**Name and contact information for the trial sponsor**	11

responsibilities: sponsor contact information			
Roles and responsibilities: sponsor and funder	#5c	**Role of study sponsor and funders, if any, in study design; collection, management, analysis, and interpretation of data; writing of the report; and the decision to submit the report for publication, including whether they will have ultimate authority over any of these activities**	11
Roles and responsibilities: committees	#5d	**Composition, roles, and responsibilities of the coordinating centre, steering committee, endpoint adjudication committee, data management team, and other individuals or groups overseeing the trial, if applicable (see Item 21a for data monitoring committee)**	11
Background and rationale	#6a	**Description of research question and justification for undertaking the trial, including summary of relevant studies (published and unpublished) examining benefits and harms for each intervention**	4-5
Background and rationale: choice of comparators	#6b	**Explanation for choice of comparators**	6-8
Objectives	#7	**Specific objectives or hypotheses**	5
Trial design	#8	**Description of trial design including type of trial (eg, parallel group, crossover, factorial, single group), allocation ratio, and framework (eg, superiority, equivalence, non-inferiority, exploratory)**	6-8
Study setting	#9	**Description of study settings (eg, community clinic, academic hospital) and list of countries where data will be collected. Reference to where list of study sites can be obtained**	6-8
Eligibility criteria	#10	**Inclusion and exclusion criteria for participants. If applicable, eligibility criteria for study centres and**	5-6

For peer review only

		**individuals who will perform the interventions (eg, surgeons, psychotherapists)**	
Interventions: description	#11a	**Interventions for each group with sufficient detail to allow replication, including how and when they will be administered**	7-8
Interventions: modifications	#11b	**Criteria for discontinuing or modifying allocated interventions for a given trial participant (eg, drug dose change in response to harms, participant request, or improving / worsening disease)**	8,11
Interventions: adherance	#11c	**Strategies to improve adherence to intervention protocols, and any procedures for monitoring adherence (eg, drug tablet return; laboratory tests)**	7-8
Interventions: concomitant care	#11d	**Relevant concomitant care and interventions that are permitted or prohibited during the trial**	7-8
Outcomes	#12	**Primary, secondary, and other outcomes, including the specific measurement variable (eg, systolic blood pressure), analysis metric (eg, change from baseline, final value, time to event), method of aggregation (eg, median, proportion), and time point for each outcome. Explanation of the clinical relevance of chosen efficacy and harm outcomes is strongly recommended**	8-10
Participant timeline	#13	**Time schedule of enrolment, interventions (including any run-ins and washouts), assessments, and visits for participants. A schematic diagram is highly recommended (see Figure)**	Figure 1
Sample size	#14	**Estimated number of participants needed to achieve study objectives and how it was determined, including clinical and statistical assumptions supporting any sample size calculations**	5
Recruitment	#15	**Strategies for achieving adequate participant enrolment to reach target sample size**	5-6
Allocation:	#16a	**Method of generating the allocation sequence (eg,**	6

For peer review only

sequence generation		**computer-generated random numbers), and list of any factors for stratification. To reduce predictability of a random sequence, details of any planned restriction (eg, blocking) should be provided in a separate document that is unavailable to those who enrol participants or assign interventions**	
Allocation concealment mechanism	#16b	**Mechanism of implementing the allocation sequence (eg, central telephone; sequentially numbered, opaque, sealed envelopes), describing any steps to conceal the sequence until interventions are assigned**	6
Allocation: implementation	#16c	**Who will generate the allocation sequence, who will enrol participants, and who will assign participants to interventions**	6
Blinding (masking)	#17a	**Who will be blinded after assignment to interventions (eg, trial participants, care providers, outcome assessors, data analysts), and how**	6-7
Blinding (masking): emergency unblinding	#17b	**If blinded, circumstances under which unblinding is permissible, and procedure for revealing a participant's allocated intervention during the trial**	N/A Clinical care team aware of allocation Page 6
Data collection plan	#18a	**Plans for assessment and collection of outcome, baseline, and other trial data, including any related processes to promote data quality (eg, duplicate measurements, training of assessors) and a description of study instruments (eg, questionnaires, laboratory tests) along with their reliability and validity, if known. Reference to where data collection forms can be found, if not in the protocol**	8-10
Data collection plan: retention	#18b	**Plans to promote participant retention and complete follow-up, including list of any outcome data to be collected for participants who discontinue or deviate from intervention protocols**	7

For peer review only

Data management	#19	**Plans for data entry, coding, security, and storage, including any related processes to promote data quality (eg, double data entry; range checks for data values). Reference to where details of data management procedures can be found, if not in the protocol**	10
Statistics: outcomes	#20a	**Statistical methods for analysing primary and secondary outcomes. Reference to where other details of the statistical analysis plan can be found, if not in the protocol**	10
Statistics: additional analyses	#20b	**Methods for any additional analyses (eg, subgroup and adjusted analyses)**	10
Statistics: analysis population and missing data	#20c	**Definition of analysis population relating to protocol non-adherence (eg, as randomised analysis), and any statistical methods to handle missing data (eg, multiple imputation)**	10
Data monitoring: formal committee	#21a	**Composition of data monitoring committee (DMC); summary of its role and reporting structure; statement of whether it is independent from the sponsor and competing interests; and reference to where further details about its charter can be found, if not in the protocol. Alternatively, an explanation of why a DMC is not needed**	11
Data monitoring: interim analysis	#21b	**Description of any interim analyses and stopping guidelines, including who will have access to these interim results and make the final decision to terminate the trial**	11
Harms	#22	**Plans for collecting, assessing, reporting, and managing solicited and spontaneously reported adverse events and other unintended effects of trial interventions or trial conduct**	11
Auditing	#23	**Frequency and procedures for auditing trial conduct, if any, and whether the process will be independent from investigators and the sponsor**	11
Research ethics	#24	**Plans for seeking research ethics committee /**	12

For peer review only

approval		**institutional review board (REC / IRB) approval**	
Protocol amendments	#25	**Plans for communicating important protocol modifications (eg, changes to eligibility criteria, outcomes, analyses) to relevant parties (eg, investigators, REC / IRBs, trial participants, trial registries, journals, regulators)**	12
Consent or assent	#26a	**Who will obtain informed consent or assent from potential trial participants or authorised surrogates, and how (see Item 32)**	6
Consent or assent: ancillary studies	#26b	**Additional consent provisions for collection and use of participant data and biological specimens in ancillary studies, if applicable**	N/A
Confidentiality	#27	**How personal information about potential and enrolled participants will be collected, shared, and maintained in order to protect confidentiality before, during, and after the trial**	10
Declaration of interests	#28	**Financial and other competing interests for principal investigators for the overall trial and each study site**	12
Data access	#29	**Statement of who will have access to the final trial dataset, and disclosure of contractual agreements that limit such access for investigators**	-
Ancillary and post trial care	#30	**Provisions, if any, for ancillary and post-trial care, and for compensation to those who suffer harm from trial participation**	-
Dissemination policy: trial results	#31a	**Plans for investigators and sponsor to communicate trial results to participants, healthcare professionals, the public, and other relevant groups (eg, via publication, reporting in results databases, or other data sharing arrangements), including any publication restrictions**	12
Dissemination policy: authorship	#31b	**Authorship eligibility guidelines and any intended use of professional writers**	-

For peer review only

Dissemination policy: reproducible research	#31c	**Plans, if any, for granting public access to the full protocol, participant-level dataset, and statistical code**	-
Informed consent materials	#32	**Model consent form and other related documentation given to participants and authorised surrogates**	-
Biological specimens	#33	**Plans for collection, laboratory evaluation, and storage of biological specimens for genetic or molecular analysis in the current trial and for future use in ancillary studies, if applicable**	N/A

The SPIRIT checklist is distributed under the terms of the Creative Commons Attribution License CC-BY-ND 3.0. This checklist can be completed online using https://www.goodreports.org/, a tool made by the EQUATOR Network in collaboration with Penelope.ai

For peer review only

CONSORT 2010 checklist of information to include when reporting a pilot or feasibility trial*

Section/Topic	Item No	Checklist item	Reported on page No
**Title and abstract**			
	1a	Identification as a pilot or feasibility randomised trial in the title	1
	1b	Structured summary of pilot trial design, methods, results, and conclusions (for specific guidance see CONSORT abstract extension for pilot trials)	2
**Introduction**			
Background and objectives	2a	Scientific background and explanation of rationale for future definitive trial, and reasons for randomised pilot trial	4-5
	2b	Specific objectives or research questions for pilot trial	5
**Methods**			
Trial design	3a	Description of pilot trial design (such as parallel, factorial) including allocation ratio	6-7
	3b	Important changes to methods after pilot trial commencement (such as eligibility criteria), with reasons	N/A
Participants	4a	Eligibility criteria for participants	5-6
	4b	Settings and locations where the data were collected	10
	4c	How participants were identified and consented	5-6
Interventions	5	The interventions for each group with sufficient details to allow replication, including how and when they were actually administered	7<br>Figure 2
Outcomes	6a	Completely defined prespecified assessments or measurements to address each pilot trial objective specified in 2b, including how and when they were assessed	8-10
	6b	Any changes to pilot trial assessments or measurements after the pilot trial commenced, with reasons	N/A
	6c	If applicable, prespecified criteria used to judge whether, or how, to proceed with future definitive trial	10-11
Sample size	7a	Rationale for numbers in the pilot trial	5
	7b	When applicable, explanation of any interim analyses and stopping guidelines	12
Randomisation:			
Sequence generation	8a	Method used to generate the random allocation sequence	6
	8b	Type of randomisation(s); details of any restriction (such as blocking and block size)	6
Allocation concealment mechanism	9	Mechanism used to implement the random allocation sequence (such as sequentially numbered containers), describing any steps taken to conceal the sequence until interventions were assigned	6

For peer review only

Implementation	10	Who generated the random allocation sequence, who enrolled participants, and who assigned participants to interventions	6
Blinding	11a	If done, who was blinded after assignment to interventions (for example, participants, care providers, those assessing outcomes) and how	7
	11b	If relevant, description of the similarity of interventions	N/A
Statistical methods	12	Methods used to address each pilot trial objective whether qualitative or quantitative	10-11
**Results**			
Participant flow (a diagram is strongly recommended)	13a	For each group, the numbers of participants who were approached and/or assessed for eligibility, randomly assigned, received intended treatment, and were assessed for each objective	Figure 1
	13b	For each group, losses and exclusions after randomisation, together with reasons	N/A
Recruitment	14a	Dates defining the periods of recruitment and follow-up	N/A
	14b	Why the pilot trial ended or was stopped	N/A
Baseline data	15	A table showing baseline demographic and clinical characteristics for each group	N/A
Numbers analysed	16	For each objective, number of participants (denominator) included in each analysis. If relevant, these numbers should be by randomised group	N/A
Outcomes and estimation	17	For each objective, results including expressions of uncertainty (such as 95% confidence interval) for any estimates. If relevant, these results should be by randomised group	N/A
Ancillary analyses	18	Results of any other analyses performed that could be used to inform the future definitive trial	N/A
Harms	19	All important harms or unintended effects in each group (for specific guidance see CONSORT for harms)	N/A
	19a	If relevant, other important unintended consequences	N/A
**Discussion**			
Limitations	20	Pilot trial limitations, addressing sources of potential bias and remaining uncertainty about feasibility	N/A
Generalisability	21	Generalisability (applicability) of pilot trial methods and findings to future definitive trial and other studies	N/A
Interpretation	22	Interpretation consistent with pilot trial objectives and findings, balancing potential benefits and harms, and considering other relevant evidence	N/A
	22a	Implications for progression from pilot to future definitive trial, including any proposed amendments	N/A
**Other information**			
Registration	23	Registration number for pilot trial and name of trial registry	-
Protocol	24	Where the pilot trial protocol can be accessed, if available	5
Funding	25	Sources of funding and other support (such as supply of drugs), role of funders	13
	26	Ethical approval or approval by research review committee, confirmed with reference number	13

For peer review only

Citation: Eldridge SM, Chan CL, Campbell MJ, Bond CM, Hopewell S, Thabane L, et al. CONSORT 2010 statement: extension to randomised pilot and feasibility trials. BMJ. 2016;355.

*We strongly recommend reading this statement in conjunction with the CONSORT 2010, extension to randomised pilot and feasibility trials, Explanation and Elaboration for important clarifications on all the items. If relevant, we also recommend reading CONSORT extensions for cluster randomised trials, non-inferiority and equivalence trials, non-pharmacological treatments, herbal interventions, and pragmatic trials. Additional extensions are forthcoming: for those and for up to date references relevant to this checklist, see www.consort-statement.org.

For peer review only

BMJ Open

Protocol for a feasibility randomised controlled trial of a musculoskeletal exercise intervention versus usual care for children with haemophilia

Journal:	*BMJ Open*
Manuscript ID	bmjopen-2019-029474.R2
Article Type:	Protocol
Date Submitted by the Author:	11-Jun-2019
Complete List of Authors:	Hashem, Ferhana; University of Kent, Centre for Health Service Studies<br>Bladen, Melanie; Great Ormond Street Hospital For Children NHS Foundation Trust, Haemophilia Centre<br>Carroll, Liz; Haemophilia Society<br>Dodd, Charlene; East Kent Hospitals University NHS Foundation Trust, Haemophilia Centre<br>Drechsler, Wendy; King's College London, School of Population Health & Environmental Sciences<br>Lowery, David; University of Kent, Centre for Health Service Studies<br>Patel, Vishal; Barts Health NHS Trust, Haemophilia Centre<br>Pellatt-Higgins, T; University of Kent, Centre for Health Services studies<br>Saloniki, Eirini; University of Kent, Centre for Health Service Studies<br>Stephensen, David; East Kent Hospitals University NHS Foundation Trust, Haemophilia Centre; Barts Health NHS Trust, Haemophilia Centre
**Primary Subject Heading**:	Haematology (incl blood transfusion)
Secondary Subject Heading:	Paediatrics, Rehabilitation medicine, Sports and exercise medicine
Keywords:	haemophilia, physiotherapy, exercise, muscle strength, physical function
Note: The following files were submitted by the author for peer review, but cannot be converted to PDF. You must view these files (e.g. movies) online.	
DOLPHIN Feasibility RCT - SPIRIT_checklist.docx	

SCHOLARONE™
Manuscripts

Protocol for a feasibility randomised controlled trial of a musculoskeletal exercise intervention versus usual care for children with haemophilia

Hashem F[1], Bladen M[2], Carroll L[3], Dodd C[5], Drechsler WI[4], Lowery D[1], Patel V[6], Pellatt-Higgins T[1], Saloniki E-C[1,7], Stephensen D[5,6]

[1]University of Kent, Centre for Health Service Studies, Canterbury, United Kingdom

[2]Great Ormond Street Hospital For Children NHS Foundation Trust, Haemophilia Centre, London, United Kingdom

Institute of Child Health, University College London, London, United Kingdom

[3]Haemophilia Society UK, London, United Kingdom

[4]Kings College London, School of Population Health & Environmental Sciences, London, United Kingdom

[5]East Kent Hospitals University NHS Trust, Haemophilia and Thrombosis Centre, Canterbury, United Kingdom

[6]Royal London Hospital, Haemophilia Centre, London, United Kingdom

[7]University of Kent, Personal Social Services Research Unit, Canterbury, United Kingdom

Corresponding Author: Dr David Stephensen, East Kent Hospitals University NHS Trust, Haemophilia and Thrombosis Centre, Canterbury, United Kingdom. david.stephensen@nhs.net

Key Words: Haemophilia, Physiotherapy, Exercise, Muscle strength, Physical function

Abstract word count: 277 words

Manuscript word count: 3283 words (excluding Figure and References)

For peer review only - http://bmjopen.bmj.com/site/about/guidelines.xhtml

ABSTRACT

Introduction: Haemophilia is a rare, inherited disorder in which blood does not clot normally, resulting in bleeding into joints and muscles. Long-term consequence is disabling joint pain, stiffness, muscle weakness, atrophy and reduced mobility. The purpose of this proposed feasibility of a randomised controlled trial (RCT) is to test the feasibility of an age-appropriate physiotherapy intervention designed to improve muscle strength, posture and the way boys use their joints during walking and everyday activities.

Methods and analysis: A small-scale two-centre RCT of a 12-week muscle strengthening exercise intervention versus usual care for young children with haemophilia will be conducted. Primary outcomes will be safety and adherence to the exercise intervention. Secondary outcomes will include: recruitment, retention and adverse event rates, clinical data, muscle strength, joint biomechanics and foot loading patterns during walking, six-minute timed walk, timed-up-and-down-stairs, EQ-5D-Y, participants' perceptions of the study, training requirements and relevant costs. Recruitment, follow-up, safety and adherence rates will be described as percentages. Participant diary and interview data will be analysed using a framework analysis. Demographic and disease variable distributions will be analysed for descriptive purposes and co-variant analysis. Estimates of differences between treatment arms (adjusted for baseline), and 75% and 95% confidence intervals will be calculated.

Ethics and Dissemination: The study has ethical approval from the London - Fulham Research Ethics Committee (17/LO/2043) as well as Health Research Authority approval. As well as informing the design of the definitive trial, results of this study will be presented at local, national and international physiotherapy and haemophilia meetings as well as manuscripts submitted to peer-reviewed journals. We will also share the main findings of the study to all participants and the Haemophilia Society.

For peer review only

ARTICLE SUMMARY

Strengths and limitations of this study

- This study investigates the feasibility of a multi-centre randomised controlled trial of a muscle strengthening exercise intervention versus usual care in children with haemophilia
- The intervention to be studied has been co-produced by children with haemophilia, their parents and healthcare professionals
- It is impossible to blind those in receipt and those delivering the intervention as to the group allocation, but we will blind the assessor as to group allocation
- At the end of this study we will not be able to answer the question "Does exercises help improve the long term health of children with haemophilia?", but we will know whether such a study could be undertaken and the best way to deliver the intervention

For peer review only - http://bmjopen.bmj.com/site/about/guidelines.xhtml

INTRODUCTION

Haemophilia is a rare, inherited disorder in which blood does not clot normally. Repeated spontaneous and traumatic bleeding into joints and muscles is the result; the ankle joint being the most common.[1] Long-term consequence is chronic arthropathy, a pattern of disabling joint pain, stiffness, muscle weakness, atrophy and reduced mobility.[2-4] The introduction of prophylaxis with clotting factor concentrates has reduced the number of bleeding episodes experienced by children, changing their lifestyle and long term outlook.[5-6] However, evidence indicates that despite prophylaxis, boys still bleed 1-2 times per year, with relatively few bleeding episodes being required to initiate bone destruction, and in some cases, articular erosion is evident with no history of joint bleeding.[1,7-9] Furthermore, children are more susceptible to blood induced damage than adults and, once established is progressive and irreversible.[10]

It is well established that muscle weakness is associated with haemophilic arthropathy in adults[4] and it is now becoming apparent that muscle strength is reduced in children, prior to the onset of clinical arthropathy.[11] In a study of 10 adolescent and adult haemophiliacs with a history of unilateral knee bleeding, Falk *et al.* (2005) found that strength of the knee extensors and flexors in haemophilic children were significantly weaker than those of the age-matched controls.[12-13] More recently, Stephensen *et al.* (2012) observed that despite normal clinical examinations and comparable levels of physical activity, young haemophilic boys with a history of ankle joint haemorrhage had significantly reduced dynamic muscle strength together with atrophy of the knee extensors, ankle dorsi- and plantarflexors when compared to age and size-matched typically developing peers.[11] Furthermore, muscle weakness of the leg is strongly correlated to muscle atrophy, reduced walking distances, slower ascent and descent of stairs, as well as reduced ankle joint motion, greater knee flexion motion and forces at the knee and ankle joint during walking.[14-15]

Therapeutic exercise is an important component of the management of other forms of arthropathy (e.g. osteoarthritis, rheumatoid arthritis) [16] and it would appear logical that exercise would be effective for people with haemophilia. There is growing evidence of the benefits of incorporating muscle strengthening exercise in their management of arthropathy.[17] It is the view of clinicians that increases in muscular strength and postural control might improve motor performance and cardiovascular fitness, limit exaggerated end-range joint movement, and

For peer review only - http://bmjopen.bmj.com/site/about/guidelines.xhtml

promote optimal transfer of weight-bearing forces through joints thereby minimising muscle imbalance, synovial impingement and associated haemarthroses or synovitis.[18] However, there is a lack of evidence to support these assumptions. A recent Cochrane Review evaluating the safety and effectiveness of exercise for people with haemophilia reported four randomly controlled studies of an exercise intervention in children with the condition and concluded the studies were of low or very low quality, due to small sample sizes and potential bias.[17] Furthermore, no paediatric study compared a muscle strengthening intervention to a control group or intervention without muscle strengthening exercises. Several studies compared two multi-component exercise interventions that included broad muscle strengthening as well as treadmill walking and cycle ergometry and it is not possible to determine the effect of muscle strengthening on muscle strength or its effect on physical function.[19-20] Furthermore, pre-adolescent and adolescent boys aged between 10 and 14 were included in these studies, and it is not known if the groups were matched for pubertal status.

Despite the apparent benefit, there is a lack of robust evidence to determine whether muscle strengthening exercise can improve or negatively affect outcomes for young children with haemophilia. The purpose of this proposed feasibility RCT is to test the feasibility of an age-appropriate physiotherapy intervention designed to improve muscle strength, posture and the way boys use their joints during walking and everyday activities.

METHODS AND ANALYSIS

The protocol version for this feasibility study is Version 2 – 19/12/2017. The Standard Protocol Items: Recommendations for Interventional Trials (SPIRIT) guidelines[21] have been used to report the study protocol.

Participants

Participants will be invited from two haemophilia centres. Twenty boys with severe or moderate haemophilia A or B with or without inhibitors on prophylactic treatment with coagulation factor concentrate, aged 6-11 years will be recruited. Boys with or without symptoms of joint damage (evaluated using the Haemophilia Joint Health Score and Haemophilia Early Arthropathy Detection with Ultrasound score) will be eligible to participate. Exclusion criteria are: von Willebrand disease, past history of fracture or trauma to the lower limb; orthopaedic surgery; acquired brain injury or any other disturbance of the central nervous system; joint or muscle

For peer review only - http://bmjopen.bmj.com/site/about/guidelines.xhtml

bleed in the lower limb in the past 6 weeks; presence of lower limb pain or unable to fully comply with verbal instructions.

Participants will be identified from each Haemophilia Centre's database of registered patients. The parents or guardians of eligible participants will be approached initially by the principle investigator at each site via telephone to provide a brief verbal explanation of the research study and ask if they would be happy to receive a patient information sheet. Should they be interested in participating in the study they will receive, by post, the patient information sheet (one for parents and a separate age appropriate version for children) at least one week prior to their next clinic appointment. This will allow for a 'cooling-off' period of a minimum of one week for the potential participants. The researcher will then be available for questions by telephone or at the participant's next clinic appointment.

At their clinic appointment, the investigator will meet with the participant and parent/ legal guardian to complete the consent form as appropriate. The investigator will further explain the study procedures, why they are being asked to consider including their child in this study and the associated risks and benefits of being included. They will then be given an opportunity to ask any further questions. The child/ parent/ legal guardian will then be asked to confirm if the potential participant agrees to take part in the study. If consent is agreed a parental consent form will be completed by the parent/ guardian and a copy will be provided. If at any point during the study process the parent/guardian does not consent to inclusion in the study then any data collected will be destroyed. Any decision to refuse or withdraw from the study will in no way influence the services that they receive. Once the child's parent/guardian has given consent, the child must also indicate that they do not object to participating in the research activity. This participant 'assent' will be sought.

<u>Study design</u>

Following written informed consent and screening of eligibility criteria, randomisation of eligible participants for the study will be performed with the online randomisation service http://sealedenvelope.com/. Utilising the secure, centralised and independent service, participants will be randomly allocated into one of two groups (Treatment Group 1 will receive a 12-week muscle strengthening exercise intervention; Treatment Group 2 will receive usual physiotherapy care for 12 weeks) on a ratio of 1:1. Allocation will be stratified by centre to ensure the same numbers of participants at each site are allocated to the two groups. The un-

For peer review only - http://bmjopen.bmj.com/site/about/guidelines.xhtml

blinded principal investigator at each site will be issued with a password to access the service to create a new randomisation for the study participants. Those allocated to Treatment Group 2 will be offered the intervention after their end of study follow-up assessment. Data will be collected prior to and immediately post intervention (Figure 1).

It is impossible to blind those in receipt and those delivering the intervention as to the group allocation. However, it will be possible – although perhaps challenging - to blind the assessor as to group allocation. Consequently, we will attempt a single blind approach in which the participant and physiotherapists delivering the intervention will be encouraged to withhold their group allocation from the assessors collecting data. We will use a range of strategies, including: instruction in the participant literature; intervention protocol and with frequent verbal prompts. In order to assess the efficacy of blinding, we will ask assessors to record which group they think participants have been allocated to at the end of each assessment. This will be presented as a binary forced choice. There are no circumstances under which it would be necessary to un-blind assessors.

<u>Intervention</u>

The intervention, developed by expert clinicians and patients utilising a modified nominal group technique is a 24-session, 12-week programme designed as 2-week progressive levels (intensity and or load) with no more than ten exercises in each session, taking 25-30 minutes to complete (Figure 2). The intervention aims to master movement control and emphasizes body-weight strength development. Pictures of the exercises and instructions for each phase will be provided in 2-week exercise diaries. Participants will be asked to complete the exercises twice per week; once with the physiotherapist who will visit the participant at home, and once supervised by their parents/ guardian. Completion of exercises, along with treatment regime, any adverse events and comments in relation to the exercise programme will be recorded in the exercise diary. They will also record participation in physical activities, including type, time and intensity. According to predetermined criteria (safe and competent), the physiotherapist will determine if it is appropriate for the participant to progress to the next exercise level. On return of the exercise diary every 2 weeks, participants will be provided with a gift voucher.

<u>Usual care</u>

Participants will continue to receive their usual care as recommended and prescribed by the haematologist, physiotherapist and other members of the healthcare team. This will include

For peer review only - http://bmjopen.bmj.com/site/about/guidelines.xhtml

factor concentrate treatment, any physiotherapy or surgical intervention or advice that is recommended by the clinical care team. There are currently no standardised treatment recommendations for children without apparent joint damage. The researchers will not make or initiate any changes to routine treatment. Any change to or initiation of new treatment or intervention will be recorded and the treating clinician will determine whether it is appropriate for the participant to continue in the study. Participants will be provided with a diary to record participation in physical activities, including type, time and intensity. Participants will be telephoned weekly to encourage dairy completion. On return of the diary every 2 weeks, participants will be provided with a gift voucher.

Outcome measures

The primary outcome measures are:

- Safety will be evaluated using annualised bleeding rates (ABR), coagulation factor usage (what the patient actually administers and records on their routine electronic diary) and reported adverse events and reactions associated with the intervention. Association with symptoms of joint disease (Haemophilia Joint Health Score and Haemophilia Early Arthropathy Detection with Ultrasound score) will be evaluated
- Adherence to the exercise intervention will be evaluated by the number of exercise session completed as a percentage of the prescribed exercise sessions. Data will be extracted from participant diaries

Secondary outcome measures are:

- Recruitment rate determined as the participants who volunteer to participate in the study as a percentage of those that are invited/ eligible
- Willingness to be randomised; evaluated at the end of study through semi-structured interviews that will be audio-recorded and be transcribed by a member of the research team
- Participant's acceptability of the trial and intervention: using semi-structured interviews, conducted at the completion of the study, the participants will be asked their views on acceptability of the trial and intervention, recruitment and exercise adherence. The interview will be audio-recorded and be transcribed by a member of the research team
- Training required to deliver the intervention and collect outcome data at study visits: using semi-structured interviews, conducted at the completion of the study, the physiotherapists will be asked their views on acceptability of the trial and intervention, recruitment and

For peer review only - http://bmjopen.bmj.com/site/about/guidelines.xhtml

exercise adherence. They will also be asked about the training requirements for delivering the intervention as well as the appropriateness of the outcomes piloted in terms of training, length of time to complete and clinical value of the data. The interview will be audio-recorded and be transcribed by a member of the research team

- Haemophilia Joint Health Score (HJHS) – a clinical examination of swelling, muscle atrophy, strength, joint crepitus, range of motion and pain at the ankle, knee and elbow joints. The HJHS is a standardised multi-item assessment, currently used in clinic as part of routine care to record musculoskeletal health[22]
- Haemophilic synovitis and arthropathy – The presence and severity of signs of haemophilic synovitis and arthropathy will be evaluated using the Haemophilia Early Arthropathy Detection with Ultrasound (HEAD-US) score. The HEAD-US protocol is a standardised scanning protocol, used to identify the presence and severity of synovitis, articular cartilage and chondral bone defects in the ankle, knee and elbow joint. The scanning procedure takes approximately 2-3 minutes per joint and we have recently shown good inter-rater repeatability of this protocol when performed by physiotherapists[23-24]
- Muscle strength of the knee extensors and ankle dorsi- and plantarflexors – Maximum torque will be recorded with a hand held dynamometer (Manual Muscle Tester, Lafayette Instrument, USA) using our standardised protocol with excellent test-retest repeatability[15]
- Joint biomechanics and motion during walking – Temporal spatial and three-dimensional (3D) joint motion during gait will be recorded using an inertial magnetic measurement gait analysis system (MVN BIOMECH Awinda, Xsens Technologies, Netherlands). Sensor units will be attached to the feet, lower legs, thighs, and pelvis with elastic straps. Motion of the sensors will be used to calculate temporal spatial and 3D lower limb joint motion
- Static and dynamic foot loading patterns – will be evaluated with a plantar pressure platform (HR Mat, Tekscan, USA). Centre of pressure motion in respect of distance and direction travelled, together with contact time as a measure of foot symmetry of foot loading for various foot segments (heel, midfoot and forefoot) along with distribution of peak pressure and pressure-time integrals will be evaluated
- Six-minute timed walk (6MTW) – Distance walked in 6 minutes. Two marker cones will be placed 30 metres apart along a corridor and each subject will be asked to walk back and forth between the cones as fast as they can for 6 minutes.[15]
- Timed up and down stairs (TUDS) – Time taken to ascend and descend a flight of 12 steps. The children will stand 30-centremetres from the bottom step and asked to walk quickly but

For peer review only - http://bmjopen.bmj.com/site/about/guidelines.xhtml

safely up the stairs, turn around on the top step and come all the way down until both feet land on the bottom step. They will be instructed to step on every step, use the hand rail if they want and not to run.[15]

- EQ-5D-Y – a five dimension (mobility, self-care, usual activities, pain/discomfort, anxiety/depression) questionnaire measuring health-related quality of life.[25]
- Costs relevant to the study – intervention costs, health and social care costs, out-of-pocket expenses and time off work costs. Healthcare costs (using National Health Service reference costs were possible) will include; training and delivering the intervention, equipment, overhead costs, visits to hospital/ haemophilia clinic, general practitioner, other healthcare services, investigations, hospitalisation, treatment, coagulation clotting factor concentrate and medication. Patient and family costs will include travel and time off work. Feasibility of collecting these data will be explored along with the resource consequences.

Data will be collected at the respective Haemophilia centres. All data collected related to the study will be confidential and anonymous. The participant will be allocated a randomised participant number code and all data collected will relate to this code. A record of which code belongs to which subject will be stored in a locked cabinet separate from the study data. Only the principle investigator (who will be a member of the participant's direct care team) will have access to this documentation. The direct care team and the principal investigator will have access to participant's personal and identifiable information during the study. Consent will be sought by the lead researcher for the research team to have access to this personal information.

Analysis methods

Recruitment, follow-up, safety and adherence rates will be described as percentages. Participant diary and interview data will be analysed to identify factors that influence these rates and acceptability of the trial using a framework analysis. Interview data from physiotherapists will be analysed using a framework analysis to determine training required to deliver the intervention and collect outcome data. Feasibility of collecting healthcare cost data will be explored along with the resource consequences. Costs will be analysed and reported using descriptive statistics. Demographic and disease variable distributions will be analysed for descriptive purposes and co-variant analysis. Walking variables will be normalised to leg length and muscle strength to body mass. Group mean and standard deviation (median and inter-quartile range where appropriate) will be calculated and reported. Change from baseline data

For peer review only - http://bmjopen.bmj.com/site/about/guidelines.xhtml

will be compared between groups at all study time points using analysis of covariance with baseline as a covariate and a factor for centre. Estimates of differences between treatment arms in the changes from baseline (adjusted for baseline), and 75% and 95% confidence intervals will be calculated. Group mean and standard deviation (median and inter-quartile range where appropriate) will be calculated pre and post intervention. These data will allow us to estimate the potential effect of the intervention and provide estimates of variability to inform the sample size required for a full RCT, as well as to refine the outcome measures by evaluating their responsiveness to changes in musculoskeletal health.

For peer review only

For peer review only - http://bmjopen.bmj.com/site/about/guidelines.xhtml

Patient and Public Involvement

Ten specialist haemophilia physiotherapists in the United Kingdom plus those experienced in sports injury were invited to take part in a Nominal Group Technique (NGT) consensus discussion to share their experience and expertise to contribute to the development of the exercise intervention. Patients' priorities, experience, and preferences informed the study design, recruitment as well as the design and delivery of the intervention. The exercise programme was demonstrated to five boys with haemophilia and their parents who were members of the Haemophilia Society, national UK patient organisation. The parents and children were asked about what they thought about the exercises and whether they could undertake them on a regular basis, where they thought the best place was for undertaking them, and how they would like to receive the information on the exercise programme. Parents and children were then asked questions about how they would feel about taking part in study testing the benefits of the exercises, issues around being allocated randomly into study groups, and then questions about what would encourage the children to continue on the exercise programme with discussions focusing upon types of rewards and incentivisation.

Management and oversight of the study

East Kent Hospital University Foundation Trust is the study sponsor. They have overall responsibility for governance of the study and have no direct role in study design, data collection, management, analysis, interpretation of data and reports and publications that arise from the study.

A Research Monitoring Committee (RMC) has been established and will meet six-monthly after the study has commenced. The RMC will be responsible for monitoring milestones and targets, data, safety, recruitment, reviewing and interpreting the results. The RMC will be chaired by an independent chair from outside the research team group. Additional meetings may be called to review any serious adverse events (SAE), adverse events (AE) and adverse reactions (AR). The Chief Investigator will: co-ordinate day to day running of the research; complete ethical approvals; communicate with recruitment sites; complete training of physiotherapists to deliver the intervention; undertake research initiation and monitoring site visits; co-ordinate data analysis and interpretation. The Chief Investigator will notify the RMC in the case of AEs and ARs. The Principal Investigator at each site will be the physiotherapist delivering the intervention. They will be responsible for recruitment and screening participants according to the inclusion and exclusion criteria, reporting AEs and ARs and local governance requirements.

For peer review only - http://bmjopen.bmj.com/site/about/guidelines.xhtml

ETHICS AND DISSEMINATION

Favourable ethical approval was obtained from the Health Research Authority and London - Fulham Research Ethics Committee and (17/LO/2043).

Study newsletters will be published by the RMC and available on the website of the Haemophilia Society. The results of this feasibility study will inform the design of a definitive RCT to test whether muscle strengthening can benefit musculoskeletal health in children with haemophilia, including whether such a study can be achieved and the best way of completing it. The results of the feasibility study will be presented at local, national and international physiotherapy and haemophilia meetings as well as manuscripts submitted to peer-reviewed journals. The main findings of the study will be shared to all participants and the Haemophilia Society.

AUTHORS CONTRIBUTION

DS, MB and WD contributed to the inception of the study. FH, MB, LC, CD, WD, DL, VP, TPH, ES and DS contributed to the study design and planning of the protocol. FH, DS, MB, WD, CD and VP contributed to the design of the intervention. FH and DS drafted the manuscript and FH, MB, LC, CD, WD, DL, VP, TPH, ES and DS critically revised and approved the manuscript.

ACKNOWLEDGEMENTS

The authors would like to acknowledge the contribution of the academic experts and specialist physiotherapists (Wendy Drechsler, Melanie Bladen, Danny Armitage, Trupti Bhandari, Lisa Geuran, Hannah Harbidge, Nicola Hubert, Ann McCarthy, Julie Sparrow, Linda Walsh), the children and parents of the Haemophilia Society for sharing their priorities, experience, and preferences that informed the study design, recruitment as well as the design and delivery of the intervention.

FUNDING:

We gratefully acknowledge the National Institute for Health Research – Research for Patient Benefit Programme for providing funds to support this project (grant number PB-PG-0215-36091). This paper reports independent research funded by the National Institute for Health Research (Research for Patient Benefit Programme, PB-PG-0215-36091). The views

For peer review only - http://bmjopen.bmj.com/site/about/guidelines.xhtml

expressed in the publication are those of the authors and not necessarily those of the NIHR or the Department of Health and Social Care.

CONFLICT OF INTEREST

The authors declare no conflicts of interest.

For peer review only

REFERENCES

1. Stephensen D, Tait R, Brodie N et al. Changing patterns of bleeding in patients with severe haemophilia A. Haemophilia 2009; 15: 1210–4.
2. Roosendaal G, van Rinsum AC, Vianen ME, et al. Haemophilic arthropathy resembles degenerative rather than inflammatory joint disease. Histopathology 1999; 34: 144–53.
3. Dunn AL. Pathophysiology, diagnosis and prevention of arthropathy in patients with haemophilia. Haemophilia, 2011; 17: 571– 8.
4. Stephensen D. Rehabilitation of patients with haemophilia after orthopaedic surgery: a case study. Haemophilia, 2005; 11(Suppl. 1): 26-29.
5. Iorio A, Marchesini E, Marcucci M, Stobart K, Chan AFC. Clotting Factor Concentrates Given to Prevent Bleeding and Bleeding-Related Complications in People with Haemophilia A or B (Review). Cochrane Libr 2011 (Issue 9). Chichester: Wiley, 2011.
6. Iorio A, Matino D, D'Amico R, Makris M. Recombinant Factor VIIa Concentrate Versus Plasma Derived Concentrates for the Treatment of Acute Bleeding Episodes in People with Haemophilia and Inhibitors (Review). Cochrane Database of Systematic Reviews (issue 8). Chichester: Wiley, 2010.
7. Feldman B, Pai M, Rivard G et al. Tailored prophylaxis in severe haemophilia A: interim results from the first 5 years of the Canadian Haemophilia Primary Prophylaxis Study. Journal of Thrombosis and Haemostasis 2006;4: 1228–36.
8. Manco-Johnson M, Abshire T, Shapiro A et al. Prophylaxis versus episodic treatment to prevent joint disease in boys with severe haemophilia. The New England Journal of Medicine 2007; 357: 535–44.
9. Scott MJ, Xiang H, Hart DP, Palmer B, Collins PW, Stephensen D, Sima CS, Hay CRM. Treatment regimens and outcomes in severe and moderate haemophilia A in the UK: The Thunder Study. Haemophilia, 2019; 25(2):205-212.
10. Pergantou H, Platokouki H, Matsinos G et al. Assessment of the progression of haemophilic arthropathy in children. Haemophilia, 2010; 16: 124-129.
11. Stephensen D, Drechsler W, Scott O. Comparison of muscle strength and in-vivo muscle morphology in young children with haemophilia and those of age-matched peers. Haemophilia 2012; 18: e302–10.
12. Falk B, Portal S, Tiktinsky R, et al. Anaerobic power and muscle strength in young hemophilia patients. Medicine Science Sports and Exercise 2000; 32: 52–7.
13. Falk B, Portal S, Tiktinsky R et al. Bone properties and muscle strength of young haemophilia patients. Haemophilia 2005; 11: 380-6.

For peer review only - http://bmjopen.bmj.com/site/about/guidelines.xhtml

14. Stephensen D, Drechsler WI & Scott OM. Influence of ankle plantar flexor muscle architecture and strength on gait in boys with haemophilia in comparison to typically developing children. Haemophilia, 2014; 20(3): 413-420.

15. Stephensen D, Taylor S, Bladen M, Drechsler W. Relationship between physical function and biomechanical gait patterns in boys with haemophilia. Haemophilia. 2016;22(6):e512-e518.

16. Geneen LJ, Moore RA, Clarke C, Martin D, Colvin LA, Smith BH.Physical activity and exercise for chronic pain in adults: an overview of Cochrane Reviews. Cochrane Database Syst Rev. 2017; 24:4:CD011279

17. Strike K, Mulder K, Michael R. Exercise for haemophilia. Cochrane Database of Systematic Reviews 2016, Issue 12. Art. No.: CD011180.

18. Blamey G, Forsyth A, Zourikian N, Short L, Jankovic N, De Kleijn P, Flannery T. Comprehensive elements of a physiotherapy exercise programme in haemophilia--a global perspective. Haemophilia. 2010;16 (Suppl 5):136-45.

19. Eid MA, Ibrahim MM, Aly SM. Effect of resistance and aerobic exercises on bone mineral density, muscle strength and functional ability in children with hemophilia. The Egyptian Journal of Medical Human Genetics (2014) 15, 139–147.

20. Mohamed RA, Sherief AEA. Bicycle ergometer versus treadmill on balance and gait parameters in children with hemophilia. The Egyptian Journal of Medical Human Genetics (2015) 16, 181–187.

21. Chan A-W, Tetzlaff JM, Altman DG, Laupacis A, Gøtzsche PC, Krleža-Jerić K, Hróbjartsson A, Mann H, Dickersin K, Berlin J, Doré C, Parulekar W, Summerskill W, Groves T, Schulz K, Sox H, Rockhold FW, Rennie D, Moher D. SPIRIT 2013 Statement: Defining standard protocol items for clinical trials. Ann Intern Med. 2013;158(3):200-207

22. Hilliard P, Funk S, Zourikian N, Bergstrom BM, Bradley CS, McLimont M, et al. Hemophilia joint health score reliability study. Haemophilia. 2006; 12(5): 518-25.

23. Martinoli C, Della Casa Alberighi O, Di Minno G, et al. Development and definition of a simplified scanning procedure and scoring method for Haemophilia Early Arthropathy Detection with Ultrasound (HEAD-US). Thrombosis and Haemostasis, 2013; 109(6): 1170-1179.

24. Stephensen D, Classey S, Harbidge H, Patel V, Taylor S, Wells A. Physiotherapist inter-rater reliability of the Haemophilia Early Arthropathy Detection with Ultrasound (HEAD-US) protocol. Haemophilia. 2018; 24(3):471-476

For peer review only - http://bmjopen.bmj.com/site/about/guidelines.xhtml

1 25. Wille N et al. (2010). Development of the EQ-5D-Y: a child-friendly version of the EQ-5D. Quality of Life Research; 19(6):887-97.

For peer review only

For peer review only - http://bmjopen.bmj.com/site/about/guidelines.xhtml

Figure Legend

Figure 1. Flowchart of participant journey through the feasibility study

Figure 2. Description of the exercise intervention

For peer review only

For peer review only - http://bmjopen.bmj.com/site/about/guidelines.xhtml

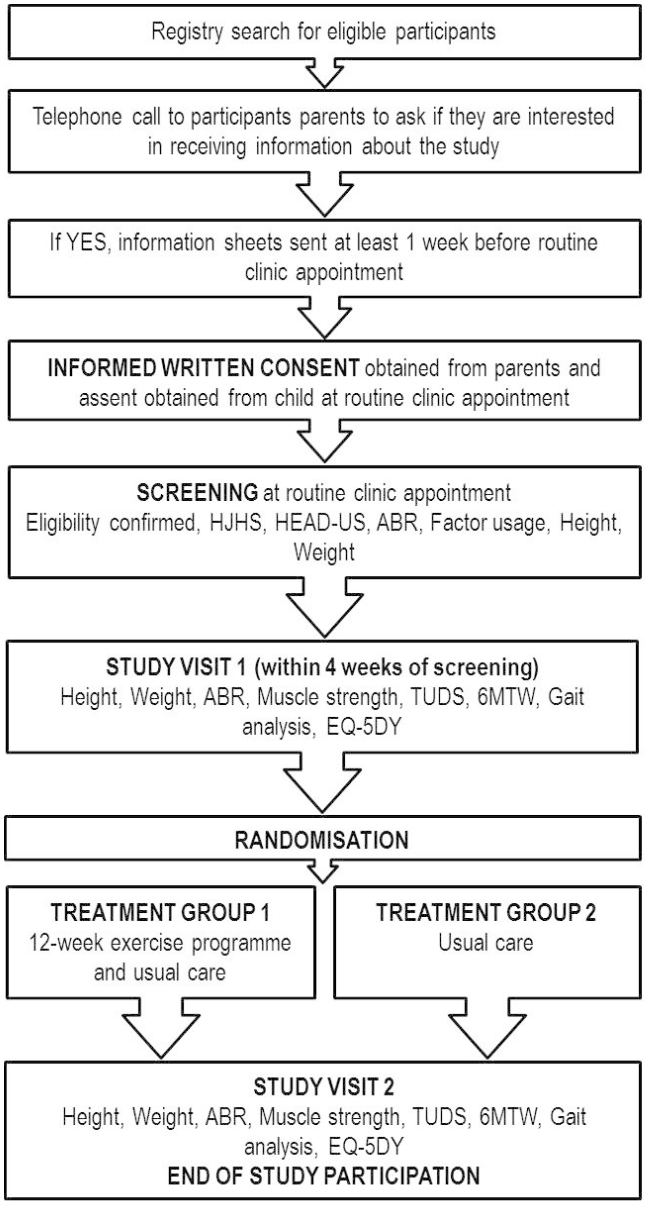

Figure 1. Flowchart of participant journey through the feasibility study

109x205mm (150 x 150 DPI)

	Stretching	Balance	Strengthening	Functional activities
**Week 1-2**	Triangle Stretch<br>Octopus Forward Stretch<br>Turtle Stretch	Tree Pose<br>Football Pose	Assisted double-leg heel raises (2 sets of 5)<br>Foot caterpillar<br>Clamshell<br>Bridge – double leg	Heel toe walk
**Week 3-4**	Triangle Stretch<br>Octopus Forward Stretch<br>Turtle Stretch	Tree pose<br>Football pose (playing hand keepy-up with a balloon)	Double-leg heel raises (2 sets of 5)<br>Foot caterpillar<br>Clamshell<br>Bridge – single leg	Heel toe walk<br>Toe heel walk
**Week 5-6**	Triangle Stretch<br>Octopus Forward Stretch<br>Turtle Stretch	Tree pose on cushion<br>Football pose on cushion	Assisted single leg heel raises (2 sets of 5)<br>Three-legged dog<br>Crab Pose	Toe heel walk<br>Step ups
**Week 7-8**	Triangle Stretch<br>Octopus Forward Stretch<br>Turtle Stretch	Tree pose on cushion<br>Football pose on cushion (playing hand keepy-up with a balloon)	Single-leg heel raises (2 sets of 5)<br>Three-legged dog<br>Forward Warrior Pose	Step ups<br>Crab walk
**Week 9-10**	Triangle Stretch<br>Octopus Forward Stretch<br>Turtle Stretch	Tree pose with eyes closed<br>Scuba diver	Single-leg heel raises (3 sets of 5)<br>Two-legged Dog<br>Forward warrior pose<br>Sideway Warrior Pose	Crab walk<br>Side step ups
**Week 11-12**	Triangle Stretch<br>Octopus Forward Stretch<br>Turtle Stretch	Tree pose on a cushion with eyes closed<br>Scuba diver on cushion	Single-leg heel raises (3 sets of 5)<br>Forward Warrior Pose<br>Sideway Warrior Pose<br>Crab Pose	Step ups<br>Side step ups

Figure 2. Description of exercise intervention

640x361mm (96 x 96 DPI)

Reporting checklist for protocol of a clinical trial.

Based on the SPIRIT guidelines.

Instructions to authors

Complete this checklist by entering the page numbers from your manuscript where readers will find each of the items listed below.

Your article may not currently address all the items on the checklist. Please modify your text to include the missing information. If you are certain that an item does not apply, please write "n/a" and provide a short explanation.

Upload your completed checklist as an extra file when you submit to a journal.

In your methods section, say that you used the SPIRIT reporting guidelines, and cite them as:

Chan A-W, Tetzlaff JM, Altman DG, Laupacis A, Gøtzsche PC, Krleža-Jerić K, Hróbjartsson A, Mann H, Dickersin K, Berlin J, Doré C, Parulekar W, Summerskill W, Groves T, Schulz K, Sox H, Rockhold FW, Rennie D, Moher D. SPIRIT 2013 Statement: Defining standard protocol items for clinical trials. Ann Intern Med. 2013;158(3):200-207

For peer review only

		Reporting Item	Page Number
Title	#1	**Descriptive title identifying the study design, population, interventions, and, if applicable, trial acronym**	1
Trial registration	#2a	**Trial identifier and registry name. If not yet registered, name of intended registry**	N/A Feasibility study
Trial registration: data set	#2b	**All items from the World Health Organization Trial Registration Data Set**	N/A
Protocol version	#3	**Date and version identifier**	5
Funding	#4	**Sources and types of financial, material, and other support**	12
Roles and responsibilities: contributorship	#5a	**Names, affiliations, and roles of protocol contributors**	12

Roles and responsibilities: sponsor contact information	#5b	**Name and contact information for the trial sponsor**	11
Roles and responsibilities: sponsor and funder	#5c	**Role of study sponsor and funders, if any, in study design; collection, management, analysis, and interpretation of data; writing of the report; and the decision to submit the report for publication, including whether they will have ultimate authority over any of these activities**	11
Roles and responsibilities: committees	#5d	**Composition, roles, and responsibilities of the coordinating centre, steering committee, endpoint adjudication committee, data management team, and other individuals or groups overseeing the trial, if applicable (see Item 21a for data monitoring committee)**	11
Background and rationale	#6a	**Description of research question and justification for undertaking the trial, including summary of relevant studies (published and unpublished) examining benefits and harms for each intervention**	4-5
Background and rationale: choice of comparators	#6b	**Explanation for choice of comparators**	6-8
Objectives	#7	**Specific objectives or hypotheses**	5
Trial design	#8	**Description of trial design including type of trial (eg, parallel group, crossover, factorial, single group), allocation ratio, and framework (eg, superiority, equivalence, non-inferiority, exploratory)**	6-8
Study setting	#9	**Description of study settings (eg, community clinic, academic hospital) and list of countries where data will be collected. Reference to where list of study sites can be obtained**	6-8

For peer review only

Eligibility criteria	#10	**Inclusion and exclusion criteria for participants. If applicable, eligibility criteria for study centres and individuals who will perform the interventions (eg, surgeons, psychotherapists)**	5-6
Interventions: description	#11a	**Interventions for each group with sufficient detail to allow replication, including how and when they will be administered**	7-8
Interventions: modifications	#11b	**Criteria for discontinuing or modifying allocated interventions for a given trial participant (eg, drug dose change in response to harms, participant request, or improving / worsening disease)**	8,11
Interventions: adherance	#11c	**Strategies to improve adherence to intervention protocols, and any procedures for monitoring adherence (eg, drug tablet return; laboratory tests)**	7-8
Interventions: concomitant care	#11d	**Relevant concomitant care and interventions that are permitted or prohibited during the trial**	7-8
Outcomes	#12	**Primary, secondary, and other outcomes, including the specific measurement variable (eg, systolic blood pressure), analysis metric (eg, change from baseline, final value, time to event), method of aggregation (eg, median, proportion), and time point for each outcome. Explanation of the clinical relevance of chosen efficacy and harm outcomes is strongly recommended**	8-10
Participant timeline	#13	**Time schedule of enrolment, interventions (including any run-ins and washouts), assessments, and visits for participants. A schematic diagram is highly recommended (see Figure)**	Figure 1
Sample size	#14	**Estimated number of participants needed to achieve study objectives and how it was determined, including clinical and statistical**	5

For peer review only

		**assumptions supporting any sample size calculations**	
Recruitment	#15	**Strategies for achieving adequate participant enrolment to reach target sample size**	5-6
Allocation: sequence generation	#16a	**Method of generating the allocation sequence (eg, computer-generated random numbers), and list of any factors for stratification. To reduce predictability of a random sequence, details of any planned restriction (eg, blocking) should be provided in a separate document that is unavailable to those who enrol participants or assign interventions**	6
Allocation concealment mechanism	#16b	**Mechanism of implementing the allocation sequence (eg, central telephone; sequentially numbered, opaque, sealed envelopes), describing any steps to conceal the sequence until interventions are assigned**	6
Allocation: implementation	#16c	**Who will generate the allocation sequence, who will enrol participants, and who will assign participants to interventions**	6
Blinding (masking)	#17a	**Who will be blinded after assignment to interventions (eg, trial participants, care providers, outcome assessors, data analysts), and how**	6-7
Blinding (masking): emergency unblinding	#17b	**If blinded, circumstances under which unblinding is permissible, and procedure for revealing a participant's allocated intervention during the trial**	N/A Clinical care team aware of allocation Page 6
Data collection plan	#18a	**Plans for assessment and collection of outcome, baseline, and other trial data, including any related processes to promote data quality (eg, duplicate measurements, training of assessors) and a description of study instruments (eg, questionnaires, laboratory tests) along with their reliability and validity, if known. Reference to**	8-10

For peer review only

		**where data collection forms can be found, if not in the protocol**	
Data collection plan: retention	#18b	**Plans to promote participant retention and complete follow-up, including list of any outcome data to be collected for participants who discontinue or deviate from intervention protocols**	7
Data management	#19	**Plans for data entry, coding, security, and storage, including any related processes to promote data quality (eg, double data entry; range checks for data values). Reference to where details of data management procedures can be found, if not in the protocol**	10
Statistics: outcomes	#20a	**Statistical methods for analysing primary and secondary outcomes. Reference to where other details of the statistical analysis plan can be found, if not in the protocol**	10
Statistics: additional analyses	#20b	**Methods for any additional analyses (eg, subgroup and adjusted analyses)**	10
Statistics: analysis population and missing data	#20c	**Definition of analysis population relating to protocol non-adherence (eg, as randomised analysis), and any statistical methods to handle missing data (eg, multiple imputation)**	10
Data monitoring: formal committee	#21a	**Composition of data monitoring committee (DMC); summary of its role and reporting structure; statement of whether it is independent from the sponsor and competing interests; and reference to where further details about its charter can be found, if not in the protocol. Alternatively, an explanation of why a DMC is not needed**	11
Data monitoring: interim analysis	#21b	**Description of any interim analyses and stopping guidelines, including who will have access to these interim results and make the final decision to terminate the trial**	11

For peer review only

Harms	#22	**Plans for collecting, assessing, reporting, and managing solicited and spontaneously reported adverse events and other unintended effects of trial interventions or trial conduct**	11
Auditing	#23	**Frequency and procedures for auditing trial conduct, if any, and whether the process will be independent from investigators and the sponsor**	11
Research ethics approval	#24	**Plans for seeking research ethics committee / institutional review board (REC / IRB) approval**	12
Protocol amendments	#25	**Plans for communicating important protocol modifications (eg, changes to eligibility criteria, outcomes, analyses) to relevant parties (eg, investigators, REC / IRBs, trial participants, trial registries, journals, regulators)**	12
Consent or assent	#26a	**Who will obtain informed consent or assent from potential trial participants or authorised surrogates, and how (see Item 32)**	6
Consent or assent: ancillary studies	#26b	**Additional consent provisions for collection and use of participant data and biological specimens in ancillary studies, if applicable**	N/A
Confidentiality	#27	**How personal information about potential and enrolled participants will be collected, shared, and maintained in order to protect confidentiality before, during, and after the trial**	10
Declaration of interests	#28	**Financial and other competing interests for principal investigators for the overall trial and each study site**	12
Data access	#29	**Statement of who will have access to the final trial dataset, and disclosure of contractual agreements that limit such access for investigators**	-
Ancillary and post trial care	#30	**Provisions, if any, for ancillary and post-trial care, and for compensation to those who suffer harm from trial participation**	-

For peer review only

Dissemination policy: trial results	#31a	**Plans for investigators and sponsor to communicate trial results to participants, healthcare professionals, the public, and other relevant groups (eg, via publication, reporting in results databases, or other data sharing arrangements), including any publication restrictions**	12
Dissemination policy: authorship	#31b	**Authorship eligibility guidelines and any intended use of professional writers**	-
Dissemination policy: reproducible research	#31c	**Plans, if any, for granting public access to the full protocol, participant-level dataset, and statistical code**	-
Informed consent materials	#32	**Model consent form and other related documentation given to participants and authorised surrogates**	-
Biological specimens	#33	**Plans for collection, laboratory evaluation, and storage of biological specimens for genetic or molecular analysis in the current trial and for future use in ancillary studies, if applicable**	N/A

The SPIRIT checklist is distributed under the terms of the Creative Commons Attribution License CC-BY-ND 3.0. This checklist can be completed online using https://www.goodreports.org/, a tool made by the EQUATOR Network in collaboration with Penelope.ai

For peer review only

CONSORT 2010 checklist of information to include when reporting a pilot or feasibility trial*

Section/Topic	Item No	Checklist item	Reported on page No
**Title and abstract**			
	1a	Identification as a pilot or feasibility randomised trial in the title	1
	1b	Structured summary of pilot trial design, methods, results, and conclusions (for specific guidance see CONSORT abstract extension for pilot trials)	2
**Introduction**			
Background and objectives	2a	Scientific background and explanation of rationale for future definitive trial, and reasons for randomised pilot trial	4-5
	2b	Specific objectives or research questions for pilot trial	5
**Methods**			
Trial design	3a	Description of pilot trial design (such as parallel, factorial) including allocation ratio	6-7
	3b	Important changes to methods after pilot trial commencement (such as eligibility criteria), with reasons	N/A
Participants	4a	Eligibility criteria for participants	5-6
	4b	Settings and locations where the data were collected	10
	4c	How participants were identified and consented	5-6
Interventions	5	The interventions for each group with sufficient details to allow replication, including how and when they were actually administered	7<br>Figure 2
Outcomes	6a	Completely defined prespecified assessments or measurements to address each pilot trial objective specified in 2b, including how and when they were assessed	8-10
	6b	Any changes to pilot trial assessments or measurements after the pilot trial commenced, with reasons	N/A
	6c	If applicable, prespecified criteria used to judge whether, or how, to proceed with future definitive trial	10-11
Sample size	7a	Rationale for numbers in the pilot trial	5
	7b	When applicable, explanation of any interim analyses and stopping guidelines	12
Randomisation:			
Sequence generation	8a	Method used to generate the random allocation sequence	6
	8b	Type of randomisation(s); details of any restriction (such as blocking and block size)	6
Allocation concealment mechanism	9	Mechanism used to implement the random allocation sequence (such as sequentially numbered containers), describing any steps taken to conceal the sequence until interventions were assigned	6

Implementation	10	Who generated the random allocation sequence, who enrolled participants, and who assigned participants to interventions	6
Blinding	11a	If done, who was blinded after assignment to interventions (for example, participants, care providers, those assessing outcomes) and how	7
	11b	If relevant, description of the similarity of interventions	N/A
Statistical methods	12	Methods used to address each pilot trial objective whether qualitative or quantitative	10-11
**Results**			
Participant flow (a diagram is strongly recommended)	13a	For each group, the numbers of participants who were approached and/or assessed for eligibility, randomly assigned, received intended treatment, and were assessed for each objective	Figure 1
	13b	For each group, losses and exclusions after randomisation, together with reasons	N/A
Recruitment	14a	Dates defining the periods of recruitment and follow-up	N/A
	14b	Why the pilot trial ended or was stopped	N/A
Baseline data	15	A table showing baseline demographic and clinical characteristics for each group	N/A
Numbers analysed	16	For each objective, number of participants (denominator) included in each analysis. If relevant, these numbers should be by randomised group	N/A
Outcomes and estimation	17	For each objective, results including expressions of uncertainty (such as 95% confidence interval) for any estimates. If relevant, these results should be by randomised group	N/A
Ancillary analyses	18	Results of any other analyses performed that could be used to inform the future definitive trial	N/A
Harms	19	All important harms or unintended effects in each group (for specific guidance see CONSORT for harms)	N/A
	19a	If relevant, other important unintended consequences	N/A
**Discussion**			
Limitations	20	Pilot trial limitations, addressing sources of potential bias and remaining uncertainty about feasibility	N/A
Generalisability	21	Generalisability (applicability) of pilot trial methods and findings to future definitive trial and other studies	N/A
Interpretation	22	Interpretation consistent with pilot trial objectives and findings, balancing potential benefits and harms, and considering other relevant evidence	N/A
	22a	Implications for progression from pilot to future definitive trial, including any proposed amendments	N/A
**Other information**			
Registration	23	Registration number for pilot trial and name of trial registry	-
Protocol	24	Where the pilot trial protocol can be accessed, if available	5
Funding	25	Sources of funding and other support (such as supply of drugs), role of funders	13
	26	Ethical approval or approval by research review committee, confirmed with reference number	13

For peer review only

Citation: Eldridge SM, Chan CL, Campbell MJ, Bond CM, Hopewell S, Thabane L, et al. CONSORT 2010 statement: extension to randomised pilot and feasibility trials. BMJ. 2016;355.
*We strongly recommend reading this statement in conjunction with the CONSORT 2010, extension to randomised pilot and feasibility trials, Explanation and Elaboration for important clarifications on all the items. If relevant, we also recommend reading CONSORT extensions for cluster randomised trials, non-inferiority and equivalence trials, non-pharmacological treatments, herbal interventions, and pragmatic trials. Additional extensions are forthcoming: for those and for up to date references relevant to this checklist, see www.consort-statement.org.

For peer review only
